# Molecular design and performance of emissive amide-containing compounds as corrosion inhibitors: synthesis, electrochemical evaluation, DFT calculations and molecular dynamics simulations†

**DOI:** 10.1039/d5ra00978b

**Published:** 2025-05-09

**Authors:** Abdelreheem A. Saddik, Mostafa Sayed, Ahmed A. K. Mohammed, Mohamed Abdel-Hakim, Mostafa Ahmed

**Affiliations:** a Department of Chemistry, Faculty of Science, Assiut University Assiut 71516 Egypt; b Innovation Incubation Center, National Tsing Hua University Hsinchu 300 Taiwan; c Chemistry Department, Faculty of Science, New Valley University El-Kharja 72511 Egypt mostafaali@mail.ustc.edu.cn mostafasayed@sci.nvu.edu.eg drmostafa@scinv.au.edu.eg; d Department of Chemistry, Faculty of Science, Al-Azhar University Assiut 71524 Egypt

## Abstract

Corrosion presents a significant challenge across various industries, resulting in considerable economic losses and safety risks. Organic compounds that contain aryl moieties and hetero atoms like nitrogen and oxygen have potential applications as efficient inhibitors and coating layers for the surface of metals. Herein, we investigate the corrosion inhibition of mild steel in 1.0 M H_2_SO_4_ using newly synthesized amide-containing compounds with naphthalene (naphthamide 6C–9C) or benzene (benzamide 6C–9C) structures. Characterization of these inhibitors *via* IR and NMR spectroscopy confirmed their chemical structures. Electrochemical analyses, including open circuit potential and potentiodynamic polarization tests, showed that these compounds significantly reduce the corrosion rate of mild steel. They achieved inhibition efficiencies up to 80% at optimal concentrations. The enhanced performance of these inhibitors is linked to their greater molecular weight and longer alkyl chains, which improve adsorption and surface coverage. Photophysical investigations revealed notable solvatochromic effects and red shifts in polar solvents, indicating strong interactions with the environment. Density Functional Theory (DFT) calculations provided further insights into the molecular structure, electronic distributions, and adsorption behavior, confirming the higher efficiency of series naphthamide 6C–9C compared to benzamide 6C–9C. Moreover, molecular Dynamics (MD) simulations corroborated the formation of stable protective layers on the metal surface. From the DFT calculations it is evidently that naphthamide 9C exhibited a smaller HOMO–LUMO energy gap compared to compound benzamide 9C, indicating higher reactivity and greater inhibitory efficiency. The integration of experimental and theoretical findings confirms that amide-containing naphthalene and benzene derivatives are highly effective corrosion inhibitors, suitable for industrial applications.

## Abstract 2

This study investigates the corrosion inhibition of mild steel in 1.0 M H_2_SO_4_ using our developed amide-containing compounds which contain naphthalene or benzene moiety, theoretical calculations were performed to support the results.

## Graphical abstract

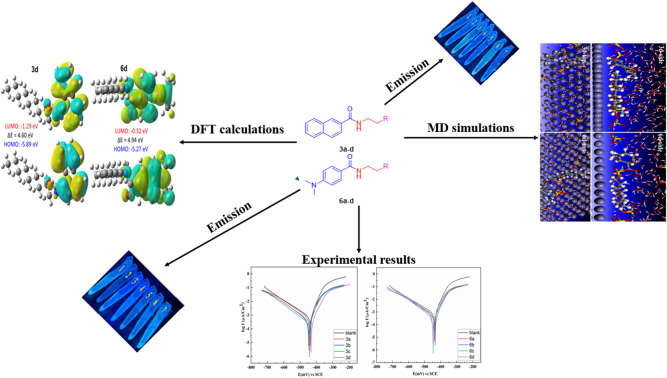

## 1. Introduction

Corrosion is a prevalent issue affecting various industries, leading to significant economic losses and safety concerns.^1–3^ Mild steel, widely used in construction, automotive, and chemical industries, is particularly susceptible to corrosion in acidic environments,^4–6^ such as sulfuric acid (H_2_SO_4_). The development of effective corrosion inhibitors is therefore critical to prolong the lifespan of mild steel and enhance its performance in corrosive environments.^7,8^ Corrosion inhibitors are substances that, when added in small concentrations, effectively reduce the corrosion rate of metals.^9^ Among the various types of corrosion inhibitors, organic compounds have gained significant attention due to their high efficiency and environmental compatibility.^10–15^ These compounds typically contain heteroatoms such as nitrogen, oxygen, and sulfur, which can donate electrons to the metal surface, forming a protective film that hinders the corrosion process.^15^

Amide-containing compounds have emerged as promising corrosion inhibitors^16–20^ due to their ability to form stable complexes with metal surfaces.^20,21^ The presence of amide groups enhances adsorption through electron donation,^22,23^ while aromatic moieties, such as naphthalene and benzene, contribute to the stability and effectiveness of the inhibition process. The synergistic effect of these functional groups can lead to the development of highly efficient corrosion inhibitors.^24,25^ Naphthalene and benzene derivatives have been extensively studied for their corrosion inhibition properties.^26–28^ Naphthalene-based compounds are known for their planar structure, which facilitates strong π–π interactions with the metal surface.^29,30^ Benzene derivatives, on the other hand, offer versatility in functionalization, allowing for the fine-tuning of their inhibitory properties.^31–34^ Incorporating naphthalene or benzene moieties with amide bond into a single compound could potentially enhance corrosion inhibition through combined adsorption mechanisms and electronic effects.^28,35^

Amide-containing aromatic compounds have garnered significant interest in the field of metal sensing due to their unique electronic and structural characteristics.^35–39^ The presence of amide groups, coupled with aromatic moieties such as naphthalene and benzene, imparts distinct photophysical properties,^40^ including strong absorption and emission profiles that are highly sensitive to their chemical environment.^41^ These properties enable the compounds to exhibit notable solvatochromism and fluorescence, making them excellent candidates for various sensing applications.^42–45^ Specifically, the ability of these compounds to form stable complexes with metal ions through coordination interactions allows them to act as effective metal sensors.^46–49^ This dual functionality of strong photophysical response and selective metal ion recognition positions amide-containing aromatic compounds as promising materials for developing advanced sensors for environmental monitoring, biological imaging, and industrial applications.^50–52^

In this study, we synthesized a series of amide-containing compounds with naphthalene and benzene moieties and evaluated their corrosion inhibition performance on mild steel in 1.0 M H_2_SO_4_. The synthesized inhibitors were characterized using various spectroscopic techniques to confirm their chemical structures. The corrosion inhibition efficiency was assessed through potentiodynamic polarization studies. Additionally, the photophysical properties of the inhibitors were investigated to understand their electronic behavior and interaction with the metal surface. To gain deeper insights into the inhibition mechanism, density functional theory (DFT) calculations were performed. DFT calculations provide valuable information on the molecular structure, electronic distribution, and adsorption behavior of the inhibitors on the mild steel surface. By correlating experimental results with theoretical calculations, we aimed to elucidate the factors contributing to the corrosion inhibition efficiency of the synthesized compounds. This comprehensive study not only highlights the potential of amide-containing naphthalene and benzene derivatives as effective corrosion inhibitors but also provides a detailed understanding of their interaction mechanisms with mild steel in acidic environments. The findings from this research could pave the way for the development of new, highly efficient, and environmentally friendly corrosion inhibitors for industrial applications.

## 2. Results and discussions

### 2.1. Synthesis of the target compounds

**Scheme 1:**
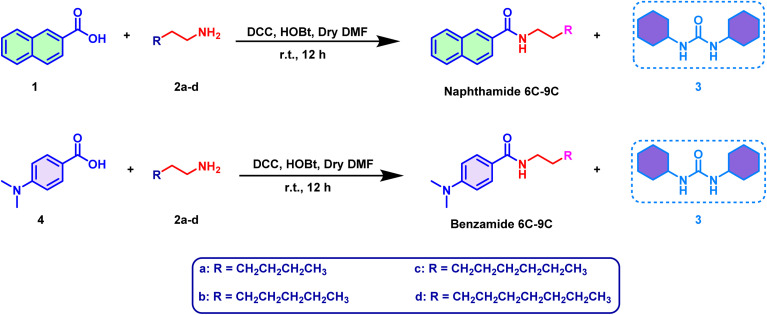
Pathway for synthesis of the target products.

The synthesis of the target amide compounds, *N*-alkyl-2-naphthamides (naphthamide 6C–9C) and 4-(dimethylamino)-*N*-alkylbenzamides (benzamide 6C–9C), was efficiently accomplished using a well-established amide coupling strategy. This process involved the reaction of either naphthanoic acid 1 or 4-dimethylamino benzoic acid 4 with various primary aliphatic amines 2a–d namely, hexyl amine, heptyl amine, octyl amine, and nonyl amine. The choice of DCC as a coupling agent and HOBt as an organic additive in dry DMF solvent provided an optimal environment for the formation of the amide bond containing products. The amide coupling reaction proceeded *via* the formation of a reactive *O*-acylisourea intermediate from the carboxylic acid in the presence of DCC. This intermediate subsequently reacts with the primary amine to form the desired amide product, releasing dicyclohexylurea 3 (DCU) as a by-product (Scheme 1). HOBt was employed to improve the reaction efficiency and reduce the formation of side products by stabilizing the *O*-acylisourea intermediate (Scheme S1†). The thermal stability of the synthesized compounds was attributed to the rigid aryl moieties present in their structures. This rigidity likely contributed to the robustness of the amide linkage, ensuring the compounds maintained their integrity under various conditions.

The obtained amide inhibitors were characterized using various spectroscopic techniques such as IR and NMR, to confirm their structures and purity. The FTIR spectra of the synthesized amides displayed characteristic absorption bands corresponding to NH and C=O functional groups. For example, compound naphthamide 6C exhibited NH stretching vibrations around 3408 cm^−1^ and 3323 cm^−1^, and a strong C=O stretching vibration at 1625 cm^−1^. Additionally, the spectra showed bands in the 2932–2858 cm^−1^ range, indicative of aliphatic C–H stretching. The ^1^H NMR spectra revealed signals attributable to both aromatic and aliphatic protons, confirming the formation of the amide bond. For instance, compound naphthamide 6C displayed a singlet at 8.61 ppm for the NH proton, along with multiplets in the aliphatic region for the CH_2_ and CH_3_ groups. Similarly, the ^13^C NMR spectra showed a distinctive signal for the carbonyl carbon (CONH) around 166.08 ppm, alongside signals for the aliphatic carbons. The structures of all other amide products naphthamide 7C–9C and benzamide 6C–9C were confirmed in the same manner with product naphthamide 6C which showed good agreement with the claimed structures. The reaction conditions employed, including room temperature and the use of dry DMF, facilitated high-yielding reactions with minimal side product formation. The white precipitate of DCU was easily removed by filtration, simplifying the purification process. Recrystallization from dilute ethanol provided the final products in high purity. The synthesis of *N*-alkyl-2-naphthamides and 4-(dimethylamino)-*N*-alkylbenzamides was effectively achieved through a DCC/HOBt-mediated coupling reaction. The process yielded structurally confirmed and thermally stable amide compounds, showcasing the reliability and efficiency of this synthetic approach for producing high-purity amides suitable for further applications. All FTIR and NMR spectra can be found in the ESI (Fig. S1–S24†).

### 2.2. Electrochemical studies

#### 2.2.1 Open circuit potential OCP

**Fig. 1:**
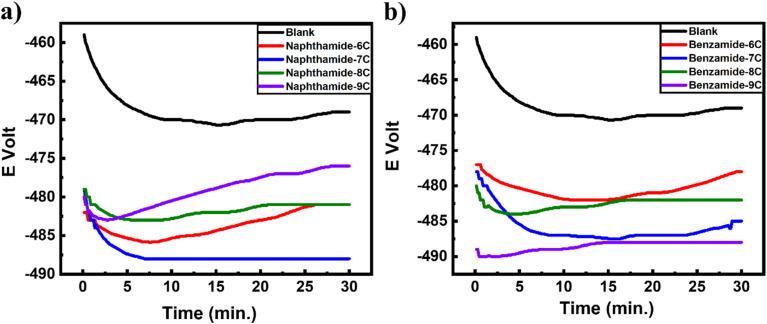
Potential–time curves of mild steel with the amid compounds: (a) naphthamide 6C–9C and b) benzamide 6C–9C.

**Fig. 2:**
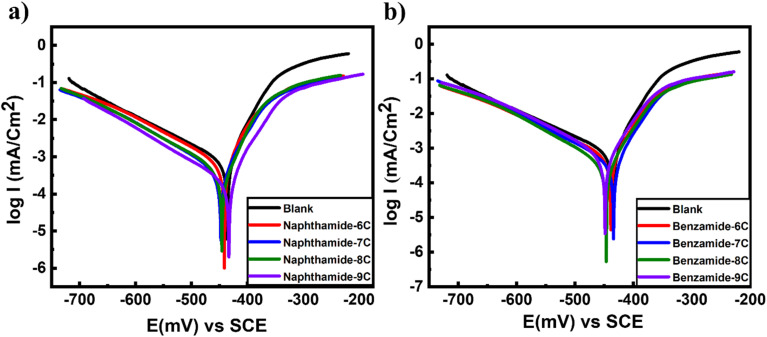
Tafel plots polarization curves of mild steel exposed to different inhibitors at 200 ppm for amide compounds: (a) naphthamide 6C–9C and (b) benzamide 6C–9C.

Fig. 1 and 2 show the curves of *E* (mV) against time (min) at zero current for MS immersed in both the blank solution and a 200 ppm concentration of the tested inhibitors (amide compounds naphthamide 6C–9C and benzamide 6C–9C). It is evident that for blank solution curves, the steady-state potential (*E*_s.s_) shifts to a more negative potential than the immersion potential *E*_im_; this shift is ascribed to the degradation of the oxide coating from the surface of the metal substrate until it attains *E*_s.s_ of the corrosion cell. When different concentrations of the testing inhibitors were added, the *E*_s.s_ value shifted more toward the positive potential than it did in the blank solution. This impact results from the creation of an adsorbed layer of inhibitor molecules on the active sites of the MS surface. Data obtained from OCP is presented in Table 1.

**Table 1:** Potential (mV) against time (min) of mild steel exposed to 1.0 M H_2_SO_4_ with amide compounds^a^

Inhibitors	−*E*_im_	−*E*_s.s_
Blank	459	469
Naphthamide 6C	482	481
Naphthamide 7C	479	488
Naphthamide 8C	479	481
Naphthamide 9C	480	476
Benzamide 6C	477	478
Benzamide 7C	478	485
Benzamide 8C	480	509
Benzamide 9C	489	488

a Where: *E*_s.s_ is the steady-state potential, *E*_im_ is the immersion potential (*E*_im_).

#### 2.2.2 Tafel polarization

Fig. 1 and 2 illustrate the potentiodynamic polarization curves displaying the corrosion of mild steel in a 1.0 M H_2_SO_4_ solution, both before and after to the addition of the examined inhibitors. The presence of amide derivatives naphthamide 6C–9C and benzamide 6C–9C induces shifts in Tafel slopes (Fig. 1 and 2). This indicated that: (i) the adsorption of inhibitor molecules on the surface of MS electrodes and (ii) the *E*_corr_ of the utilized inhibitors varies positively from that of the blank solution, with the difference not exceeding 85 mV, thereby demonstrating that these inhibitors are mixed-type, resulting in a reduction of both anodic and cathodic Tafel slopes.^53^Table 2 presents the metrics derived from TF, including *I*_corr_, *E*_corr_, CR, IE%, and *θ* of MS, both with and without inhibitors. In the absence of analyzed inhibitors, *I*_corr_ rises to 2699 (μA cm^−2^) and CR ascends to 2488 mpy. Furthermore, Fig. 2 shows the Tf plots of the effect of amide compounds (200 ppm of naphthamide 6C–9C and benzamide 6C–9C) on the mild steel in 1.0 M sulfuric acid. Compounds (naphthamide 6C–9C) give close corrosion inhibition, and the same behavior is exhibited by compounds (benzamide 6C–9C) with corrosion inhibition in the range of 80% and 75% respectively (Table 2). The higher inhibition efficiency of (naphthamide 6C–9C) than (benzamide 6C–9C) compounds attributed to the higher molecular weight of the compounds (naphthamide 6C–9C) compared to (benzamide 6C–9C) compounds and the chain length plays a role in the inhibition causing a slight change among compounds for every group, which promotes higher inhibition and higher surface area coverage of the metal.^54–56^ These results completely agreed with the results of theoretical quantum chemistry calculations that were performed, which investigated that there is no difference in the energy gap between compounds in the same group (naphthamide 6C–9C and benzamide 6C–9C). Moreover, the observed trend in inhibition efficiency can be interpreted by a precious look over structural aspects of investigated compounds. Generally, all naphthalene moiety containing inhibitors exhibit a greater inhibition efficiency than that of corresponding phenyl derived inhibitors. This might be attributed to the fact that naphthalene moiety has an extended planar aromatic system with delocalized pi electrons, this contributes to greater interaction with metallic iron, resulting in a greater surface coverage and hence greater protection. Meanwhile, the hydrophobic tail can be oriented towards the solution which might repel polar corrosive species from further attack on metal surface.

**Table 2:** Potentiodynamic polarization parameters of mild steel immersed in 1.0 M H_2_SO_4_ with amide compounds

Inhibitors	*I* (μA cm^−2^)	C.R	IE%	*θ*
Blank	2699	2488.57	—	—
Naphthamide 6C	576.2	531.277	80.316	0.8032
Naphthamide 7C	574.1	529.341	80.388	0.8039
Naphthamide 8C	573.1	528.419	80.422	0.8042
Naphthamide 9C	570.2	525.745	80.521	0.8052
Benzamide 6C	724.9	668.384	75.236	0.7524
Benzamide 7C	723.4	667.001	75.287	0.7529
Benzamide 8C	720.2	664.05	75.396	0.754
Benzamide 9C	719.1	663.036	75.434	0.7543

As shown in Table S1,† our one-step synthesized amides demonstrate inhibition efficiency comparable to that of various established corrosion inhibitors. Unlike these alternatives, which often require multi-step syntheses and costly solvents, our amides offer a more economical option without compromising protective performance. Considering the advantages of the ease synthesis and one-pot strategy of our current study over the other reported methods, this technique offers a straightforward and effective strategy for the obtaining of new inhibitors.

### 2.3. Photophysical study

**Fig. 3:**
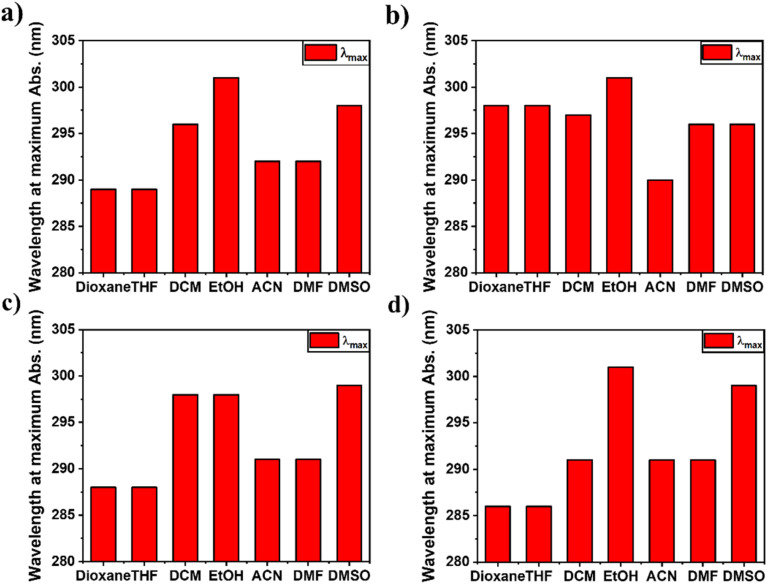
(a–d) The relation between the wavelength at maximum absorption of compounds 6a–d at different organic solvents (dioxane, THF, DCM, EtOH, ACN, DMF, and DMSO) at 100 μM; (a) benzamide 6C, (b) benzamide 7C, (c) benzamide 8C, and (d) benzamide 9C.

The absorption spectra of compounds naphthamide 6C–9C and benzamide 6C–9C were recorded in various solvents (DCM, EtOH, THF, dioxane, ACN, DMF, and DMSO) at room temperature. The absorption maxima of compounds naphthamide 6C–9C in various solvents at concentration 100 μM showed *λ*_max_ at 281 nm confirmed with the absorption maxima of the naphthalene moiety in different organic solvents with little red-shift to 2–3 nm by changing the solvents (Fig. S24†).^57^ Furthermore, compounds benzamide 6C–9C at concentration 100 μM exhibited an absorption band at *λ*_max_ = 287 nm in THF, characteristic of the 4-dimethylaminobenzamide moiety.^58^ However, these compounds displayed a solvatochromic effect in their absorption spectra, with a red shift observed in various solvents ranging from 289 nm to 294 nm and reaching 301 nm in EtOH, which can be attributed to the increased stabilization of the excited state relative to the ground state by polar solvents. This reduction in the energy gap between the excited and ground states leads to the observed bathochromic shift (Fig. 3, S24 and S25†).^59^

**Fig. 4:**
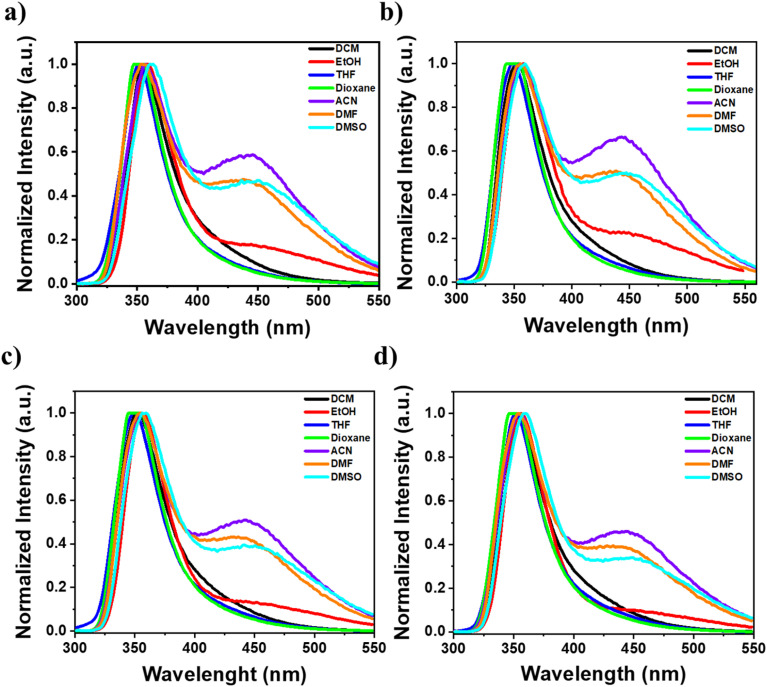
(a–d) Normalized emission spectra of compounds 6a–d at different organic solvents (DCM, EtOH, THF, dioxane, ACN, DMF, and DMSO) at 100 μM (*λ*_ex_ = 287 nm); (a) benzamide 6C, (b) benzamide 7C, (c) benzamide 8C, and (d) benzamide 9C.

Furthermore, the fluorescence emission of compounds naphthamide 6C–9C and benzamide 6C–9C was examined in various organic solvents. Compounds naphthamide 6C–9C exhibited a red shift in their emission spectra, ranging from 350 to 355 nm compared to the 334 nm emission in THF, as solvent polarity increased. Notably, these compounds displayed the strongest emission in ethanol. Similarly, compounds benzamide 6C–9C demonstrated a red shift in emission spectra (351–363 nm) compared to dioxane (348 nm), with pronounced fluorescence. Interestingly, dual emission at 450 nm was observed in more polar solvents (ethanol, acetonitrile, DMF, and DMSO). This phenomenon could be attributed to either an anomalous emission transition from a dark state or the limited solubility of these compounds in these solvents, especially in acetonitrile (Fig. 4, S26–S28†).^60^ Further, the concentration-dependent emission spectra of compounds naphthamide 6C–9C were examined in ethanol, while compounds benzamide 6C–9C were evaluated in dioxane at different concentrations (5, 10, 50, and 100 μM). As the concentration increased, we observed a corresponding increase in fluorescence intensity without any shift in the emission spectra. This suggests that the compounds' fluorescence properties remain unchanged by concentration changes (Fig. S29†).

### 2.4. Quantum-chemical calculations

#### 2.4.1 DFT and MD simulations

**Fig. 5:**
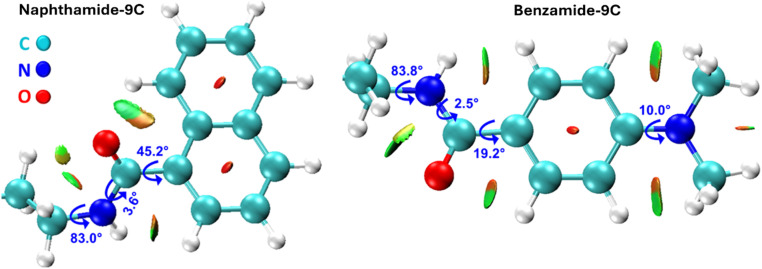
Optimized structures of compounds naphthamide 9C and benzamide 9C calculated at the B3LYP-D3(BJ)/6-31G(d) level. Key structural parameters are annotated in the figure. Intramolecular interactions, analyzed using the Multiwfn program, are based on geometries optimized at the same computational level. van der Waals interactions are represented by green surfaces.

Density functional theory (DFT) calculations were conducted on the eight compounds to elucidate their geometric and electronic structures, provide a molecular-level interpretation of the experimental findings, and uncover the inhibitory mechanisms of molecules naphthamide 9C and benzamide 9C on the iron (Fe) surface. Fig. 5 displays the optimized structures of compounds naphthamide 9C and benzamide 9C at the B3LYP-D3(BJ)/6-31G(d) level, accompanied by reduced density gradient (RDG) plots. These plots are instrumental in identifying noncovalent interactions. Selected geometric parameters and intramolecular forces are also illustrated. For clarity, the full straight-chain alkane segments of both compounds are not fully depicted. In compound naphthamide 9C, the torsional angle between the amide group and the naphthalene ring is 45.2°. The green surfaces between the amide group, naphthalene ring, and alkane chain indicate the presence of van der Waals interactions. Similarly, in compound benzamide 9C, the torsional angle between the amide group and the dimethylaniline moiety is 19.2°, with van der Waals forces governing the intramolecular interactions. The remaining molecules exhibit comparable geometric parameters, as they differ primarily in the length of their alkane side chains.

**Fig. 6:**
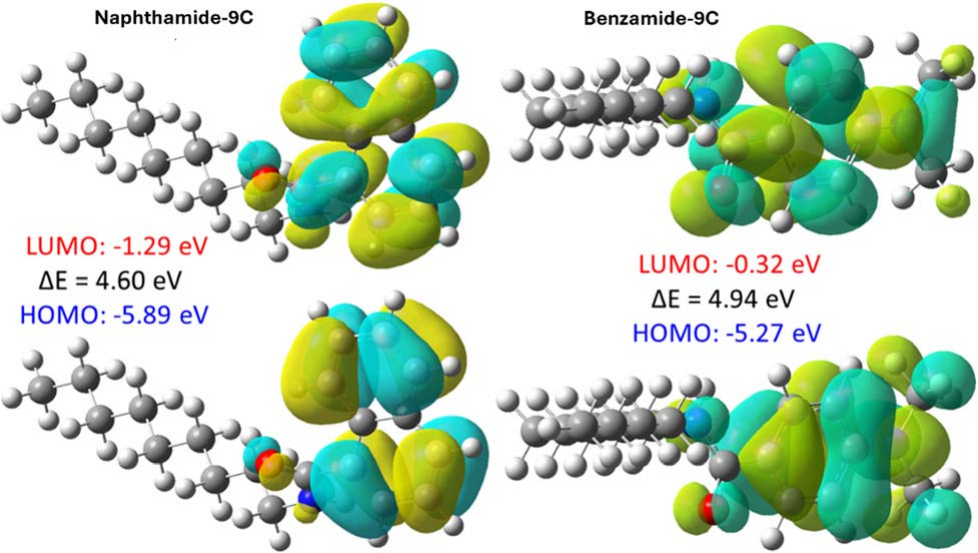
Distribution of the HOMO and LUMO for compounds naphthamide 9C and benzamide 9C, along with their corresponding energies, computed at the B3LYP-D3(BJ)/6-31G(d) level.

Next, the isosurface maps of the highest occupied molecular orbital (HOMO) and lowest unoccupied molecular orbital (LUMO) were computed for all structures at the B3LYP-D3(BJ)/6-31G(d) level to elucidate the electronic structure of these compounds. These calculations reveal the orbital distributions, which are critical for understanding the interaction between the inhibitor molecules and the iron (Fe) surface. The frontier molecular orbitals (FMOs) play a key role in this interaction, as electrons are transferred from the HOMO of the inhibitor to the vacant orbitals of the metal, while the LUMO of the inhibitor accepts electrons from the filled d-orbitals of the metal. Fig. 6 illustrates the HOMO and LUMO distributions, along with their energy differences, for compounds naphthamide 9C and benzamide 9C. For compound naphthamide 9C, both the HOMO and LUMO are localized over the amide group and the naphthalene ring. The HOMO primarily consists of the lone pairs of oxygen (O) and nitrogen (N) atoms, as well as the π orbitals of the aromatic ring, while the LUMO is composed of the π* orbitals of the aromatic system. The aliphatic side chain does not contribute to either orbital. The HOMO energy is −5.89 eV, the LUMO energy is −1.29 eV, and the HOMO–LUMO gap is 4.60 eV. Compounds naphthamide 6C–8C exhibit similar orbital distributions and identical energy gaps to naphthamide 9C, as they differ only in the length of the alkane tail. Consequently, their orbital distributions are not explicitly shown. Similarly, for compound benzamide 9C, both the HOMO and LUMO are localized on the dimethylaniline moiety and the amide group, with no contribution from the aliphatic tail. The HOMO, LUMO, and energy gap for 6d are −5.27 eV, −0.32 eV, and 4.94 eV, respectively. These results align with chemical intuition. In both molecules, the conjugated system, along with the amide group, serves as the most effective electron-donating and electron-withdrawing region. During interactions with the Fe surface, the amide group and the aromatic system are anticipated to be the most reactive sites.

**Fig. 7:**
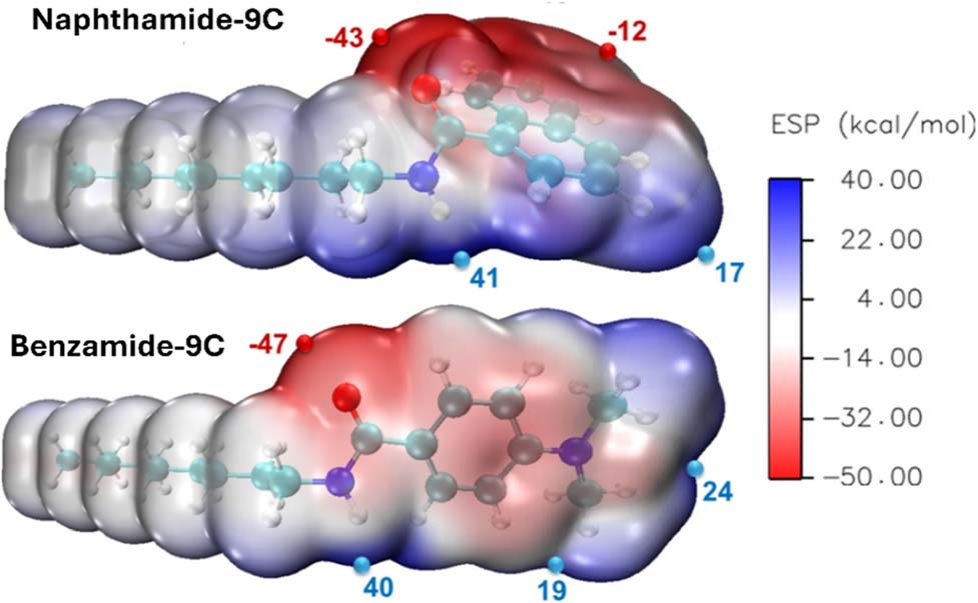
Molecular electrostatic potential (MESP) surface map of compounds naphthamide 9C and benzamide 9C, displaying electrostatic potential maxima (positive values) and minima (negative values), calculated at the B3LYP-D3(BJ)/6-31G(d) level.

Furthermore, the molecular electrostatic potential (MESP) map is a widely utilized tool for identifying nucleophilic and electrophilic sites within a molecule. On the MESP map, regions of low electron density (electrophilic sites) are represented by blue coloration, while regions of high electron density (nucleophilic sites) are indicated by red coloration. Neutral regions are depicted in white. Fig. 7 presents the MESP analysis of compounds naphthamide 9C and benzamide 9C, highlighting surface maxima and minima. The maxima correspond to points with the highest positive electrostatic potential (ESP), whereas the minima represent points with the most negative ESP. This analysis identifies the most electrophilic and nucleophilic regions within the molecules. In both compounds, the highest negative potential is localized around the carbonyl group, with values of −43 kcal mol^−1^ for naphthamide 9C and −47 kcal mol^−1^ for benzamide 9C. Compound benzamide 9C exhibits slightly greater nucleophilicity compared to naphthamide 9C, attributed to the extended conjugation in the naphthalene ring, which distributes electron density and reduces the electron density around the carbonyl group. The red coloration around the aromatic rings in both molecules arises from the presence of π electrons. The most electrophilic region in both molecules is the hydrogen atom of the amide group, with a positive ESP of approximately 40 kcal mol^−1^. These findings indicate that the carbonyl (C=O) and amide N–H groups are the primary nucleophilic and electrophilic sites, respectively. Consequently, interactions with the metal surface are expected to predominantly involve these two functional groups and the aromatic system, with minimal contribution from the aliphatic side chain.

#### 2.4.2 Formation of a protective coat

**Fig. 8:**
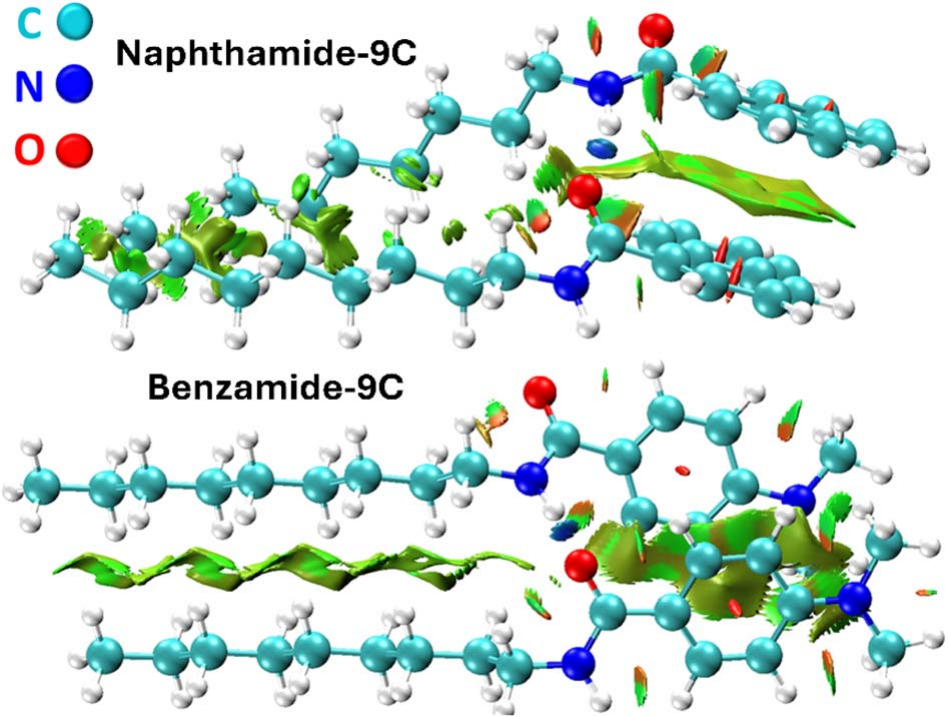
The optimized geometry of the dimers of molecules naphthamide 9C and benzamide 9C optimized at the B3LYP-D3(BJ)/6-31G(d) level. Intermolecular interactions are depicted as follows: blue color indicates a hydrogen bond, green surface for van der Waals interactions, and red for rings.

To date, our investigation has focused solely on individual isolated compounds, neglecting the role of intermolecular forces. Strong intermolecular interactions can cause molecules to aggregate into dimers or larger assemblies, which can form a protective layer on the metal surface, shielding it from corrosive agents. The stability and effectiveness of this protective layer are directly influenced by the strength of the intermolecular forces between the inhibitor molecules. The DFT calculations, incorporating dispersion corrections, are a widely used method for studying noncovalent interactions between molecules. In this part, DFT was employed to examine the attractive forces between inhibitor molecules and their aggregation propensity. Neither inhibitor exhibits intramolecular hydrogen bonding. As shown in Fig. 5, the only intramolecular interactions present are van der Waals forces. However, both compounds can form intermolecular hydrogen bonds *via* the amide group, facilitating the formation of an extended molecular layer on the metal surface. To investigate this, geometry optimization of the dimers of naphthamide 9C and benzamide 9C was performed using DFT at the same computational level. Multiple binding geometries were evaluated, and those with the strongest binding energies were selected. Fig. 8 illustrates the optimized geometries and reduced density gradient (RDG) plots of the dimers for both inhibitors. The analysis reveals the formation of a single hydrogen bond between the two molecules, represented by a blue surface between the oxygen atom of one molecule and the N–H group of the other. Additionally, significant van der Waals interactions are observed between the molecules in the dimer. The calculated binding energies for the dimers are 60.8 kcal mol^−1^ for naphthamide 9C and 59.4 kcal mol^−1^ for benzamide 9C, indicating a strong tendency for both inhibitors to aggregate and form a stable protective layer on the metal surface. The dimer of naphthamide 9C is more stable than that of benzamide 9C, primarily due to stronger π–π stacking interactions facilitated by the naphthalene ring in naphthamide 9C, compared to the benzene ring in benzamide 9C. These results suggest that naphthamide 9C may exhibit slightly superior inhibitory performance compared to benzamide 9C.

The global reactivity descriptors for compounds naphthamide 9C and benzamide 9C were computed using DFT calculations to evaluate their reactivity. These descriptors were derived from the energies of HOMO and LUMO.^61^ Generally, molecules with higher HOMO energies (*E*_HOMO_) exhibit enhanced electron-donating capabilities, while lower LUMO energies (*E*_LUMO_) indicate a greater capacity to accept electrons from the metal surface. Consequently, inhibitors with high *E*_HOMO_ and low *E*_LUMO_ values are expected to exhibit stronger interactions with the metal surface and higher inhibition efficiency. The energy gap (Δ*E*) between *E*_HOMO_ and *E*_LUMO_ provides insight into the inhibitor's efficiency, with smaller gaps correlating with higher reactivity and better inhibitory performance. Table 3 presents the calculated global reactivity descriptors for compounds naphthamide 9C and benzamide 9C. Both inhibitors exhibit comparable ionization potentials (IP = −*E*_HOMO_) and electron affinities (EA = −*E*_LUMO_), with nearly identical HOMO–LUMO energy gaps. Molecules with low IP (high *E*_HOMO_) are strong electron donors, while those with low EA (low *E*_LUMO_) are effective electron acceptors. Compound benzamide 9C, with a higher *E*_HOMO_, demonstrates superior electron-donating ability compared to naphthamide 9C, whereas naphthamide 9C, with a lower *E*_LUMO_, acts as a better electron acceptor. Additionally, compound naphthamide 9C exhibits slightly higher reactivity due to its smaller energy gap (Δ*E*). These results align with the molecular electrostatic potential (MESP) analysis shown in Fig. 7, where compound naphthamide 9C displays the most electrophilic region and benzamide 9C the most nucleophilic region. A smaller Δ*E* indicates greater polarizability and a stronger tendency to adsorb onto the metal surface, suggesting that naphthamide 9C is a more effective inhibitor than benzamide 9C. Further, chemical hardness (*η*) is inversely related to chemical reactivity, with higher hardness values corresponding to lower reactivity and reduced inhibitory effectiveness. Compound naphthamide 9C exhibits lower hardness compared to benzamide 9C, further supporting its higher reactivity. The electronegativity (*χ*) of benzamide 9C is 2.79 eV, which is 0.8 eV lower than that of naphthamide 9C, indicating that naphthamide 9C has a greater ability to withdraw electrons from the filled d-orbitals of iron (Fe) and adhere more strongly to the metal surface. In summary, the calculated parameters—including HOMO, LUMO, Δ*E*, chemical hardness, electronegativity, and maximum charge transfer are consistent and collectively indicate that naphthamide 9C is a slightly more efficient inhibitor for Fe than benzamide 9C. These computational findings agree with experimental observations.

**Table 3:** Calculated properties for molecules naphthamide 9C and benzamide 9C and their protonated forms (naphthamide 9C–H and benzamide 9C–H) at the B3LYP-D3(BJ)/6-31G(d) level, including dipole moment (Debye), energies of the HOMO and LUMO, HOMO–LUMO energy gap (Δ*E*), ionization potential (IP), electron affinity (EA), chemical hardness (*η*), chemical potential (*μ*), electronegativity (*χ*), electrophilicity index (*ω*), and maximum charge transfer (Δ*N*_max_). All quantities, except dipole moments, are reported in electron volts (eV)

Comp.	Dipole	*E*_HOMO_	*E*_LUMO_	Δ*E*	IP	EA	*η*	*μ*	*χ*	*ω*	Δ*N*_max_
Naphthamide 9C	3.2	−5.89	−1.29	4.60	5.89	1.29	2.30	−3.59	3.59	2.80	1.56
Benzamide 9C	4.9	−5.27	−0.32	4.94	5.27	0.32	2.47	−2.79	2.79	1.58	1.13
Naphthamide 9C–H	12.3	−9.58	−5.73	3.85	9.58	5.73	1.93	−7.65	7.65	15.20	3.97
Benzamide 9C–H	14.9	−8.93	−5.39	3.54	8.93	5.39	1.77	−7.16	7.16	14.49	4.05

#### 2.4.3 Protonated forms

Both naphthamide 9C and benzamide 9C have O and N atoms. These heteroatoms are known for their tendency to be protonated in acidic media. Molecule naphthamide 9C has two possible sites of protonation: O and N atoms. Fig. 7 suggests that the O atom is the most nucleophilic region in the molecule because it has the highest negative potential. Thus, the O atom is the best site for protonation. To reveal the site of protonation, geometry optimization of the O- and N-protonated forms of naphthamide 9C was performed and the energy of both structures was calculated using DFT. The O-protonated form was more stable by 11.1 kcal mol^−1^. According to Maxwell–Boltzmann distribution, such a high energy difference indicates that only the O-protonated form exists. benzamide 9C, on the other hand, has three possible sites of protonation: two nitrogen and one oxygen atom. DFT calculations showed that the O-protonated form is more stable than the other two by more than 9 kcal mol^−1^. Moreover, Table 3 shows a significant decrease in the energy of both HOMO and LUMO of both molecules upon protonation. The rest of the reactivity descriptors change profoundly as well. Thus, both forms are expected to show different behavior. These results confirm the importance of studying the electronic structure of the protonated forms in addition to the neutral ones. The neutral forms are better electron donors, while the protonated ones are better acceptors. Both protonated forms are more polar and more chemically reactive (have smaller Δ*E*) than the neutral ones. The electronegativity of the neutral naphthamide 9C and benzamide 9C are 3.59 and 2.79 eV. The electronegativity of Fe (110) is 4.82 eV.^62^ Fe is more electronegative than both neutral inhibitors, and thus electrons will transfer from the inhibitor to the metal surface. The electronegativity of the protonated forms, on the other hand, are much higher than that of Fe. This reflects the high tendency of both protonated forms to attract the electrons of the d-orbital from the metal surface. The protonated form of naphthamide 9C has a higher electronegativity than benzamide 9C, and thus will have a stronger interaction with Fe. The amount of electrons transferred from the surface of the metal to the protonated inhibitor can be calculated from the Pearson theory as:^63^ Δ*N* = (*χ*_inhibitor_ − *χ*_Fe_)/2(*η*_inhibitor_ + *η*_Fe_).

Δ*N* for naphthamide 9C and benzamide 9C are 0.57 and 0.51 electrons. These numbers show that both inhibitors have strong interactions (charge transfer) with the surface of the metal and naphthamide 9C has a slightly higher inhibition efficiency than benzamide 9C.

#### 2.4.4 MD simulations

**Fig. 9:**
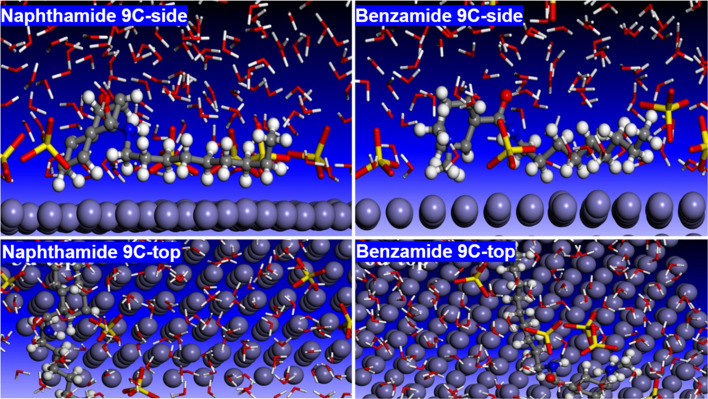
Side and top views of the adsorption of naphthamide 9C and benzamide 9C on the Fe (110) surface in aqueous solution at the end of MD simulations of 1 ns.

Molecular dynamics (MD) simulations were conducted to study the dynamic behavior of both inhibitors on the iron (Fe) surface. A simulation box was constructed containing a Fe slab, an inhibitor molecule, sulfuric acid, and water molecules. Fig. 9 illustrates the top and side views of the adsorption configurations of compounds naphthamide 9C and benzamide 9C on the Fe surface after 1 nanosecond (ns) of MD simulations. The results reveal that, as the simulation progresses, the inhibitor molecules adopt a nearly horizontal orientation on the metal surface. This configuration effectively blocks access of water and sulfuric acid molecules to the metal surface, as demonstrated in the side views of Fig. 9. Strong interactions between the inhibitor molecules and the Fe surface anchor the inhibitors in place, while the hydrophobic nature of the long alkyl chains prevents the inhibitors from migrating into the hydrophilic solvent. These findings confirm the formation of a protective adsorbed layer of inhibitors on the Fe surface, shielding it from corrosive attack by the acidic medium. Both DFT calculations and MD simulations indicate that compounds naphthamide 9C and benzamide 9C are effective inhibitors for Fe. The key functional regions in both molecules are the amide group and the aromatic rings, which correspond to the locations of the HOMO (electron-donating region) and LUMO (electron-accepting region). These groups facilitate strong interactions with the Fe surface through electron donation and acceptance. Additionally, the hydrophobic alkyl chains enhance the inhibitors' affinity for the metal surface by repelling them from the polar solvent. This aligns with experimental observations, as inhibitors with longer alkyl chains exhibit higher inhibition efficiency compared to those with shorter chains.

### 2.5. Mechanism of corrosion inhibition

**Scheme 2:**
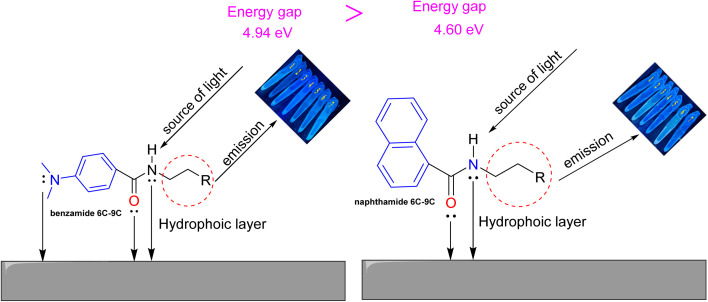
Suggested mechanism for the adsorption of *N*-alkyl-2-naphthamides (naphthamide 6C–9C) and 4-(dimethylamino)-*N*-alkylbenzamides (benzamide 6C–9C) on the surface of iron metal.

As outlined in Scheme 2. The adsorption of investigated amides can be attributed to the presence of amide group which consider as electron donations source such as nitrogen and oxygen and aryl group which have π-electrons of aromatic ring.^64,65^ The mechanism displays the interaction between the amides and the surface of the metal through coordination bond between unshared electrons of both oxygen and nitrogen atoms as well as π-electrons of both naphthalene and benzene moieties. In addition, the presence of alkyl group can form a hydrophobic layer which able to be coated on the metal surface^66^. Interestingly, all the synthesized amides exhibited emission behavior in different solvents which can form emissive layer as indicator for the coated layer of the inhibitors. DFT calculations confirmed the suggested mechanism, while Fe is more electronegative than both neutral inhibitors, and thus electrons will transfer from the inhibitor to the metal surface. The regions with the highest electron density (highest negative potential on Fig. 3) will have the strongest interaction with the Fe surface. Namely, the lone pairs of oxygen and nitrogen. However, the protonated inhibitor will accept electrons from metal to their LUMO orbital.

## 3. Conclusion

This research involved the synthesis and characterization of two series of amide compounds, *N*-alkyl-2-naphthamides (naphthamide 6C–9C) and 4-(dimethylamino)-*N*-alkylbenzamides (benzamide 6C–9C), through a DCC/HOBt-mediated coupling reaction. The synthesized amides demonstrated notable corrosion inhibition capabilities for mild steel in a 1.0 M H_2_SO_4_ solution, with compounds naphthamide 6C–9C achieving inhibition efficiencies of up to 80%. The superior performance of naphthamide 6C–9C is linked to their higher molecular weights and extended alkyl chains, which enhance surface coverage and adsorption on the metal. These conclusions were reinforced by significant shifts in corrosion potential and reductions in corrosion current densities. Furthermore, photophysical analyses revealed pronounced solvatochromic effects in the compounds. The DFT calculations offered deeper insights into the electronic characteristics and adsorption mechanisms of the inhibitors. Compound naphthamide 9C has a smaller HOMO–LUMO energy gap than benzamide 9C, which indicates that it is more reactive and is a more efficient inhibitor. DFT calculations also showed that naphthamide 9C is better in forming a protective layer on the surface of the metal than benzamide 9C. Molecular dynamics (MD) simulations showed the tendency of both inhibitors to attached itself to the surface of the metal and protect it from the corrosive assault of the acid. Both experimental and theoretical studies consistently demonstrate that compounds naphthamide 6C–9C are more effective corrosion inhibitors than compounds 6a–d. The experimental data showing higher inhibition efficiencies for naphthamide 6C–9C are supported by the theoretical findings of more favorable electronic properties and better adsorption behavior.

## 4. Experimental

### 4.1. General procedures for synthesis of *N*-alkyl-1-naphthamides (naphthamide 6C–9C)

A mixture of 2-naphthoic acid (1 g, 5.80 mmol) and primary aliphatic amine (1 equiv., 5.80 mmol) were dissolved in DMF (10 ml) then DCC (1.2 equiv., 1.438 g, 6.96 mmol) and 1-hydroxybenzotriazole (1.11 equiv., 0.941 g, 6.44 mmol) were added, then the resulting solution was stirred for 12 h at room temperature. A white precipitate formed, which was filtered off and the remaining solution was poured onto ice-water mixture, the formed precipitate was collected, dried, and recrystallized from diluted ethanol.

#### 4.1.1 *N*-hexyl-2-naphthamides (naphthamide 6C)

A white precipitate was obtained in 80% yield. ^1^H NMR (400 MHz, DMSO-d_6_) *δ* (ppm): 0.87 (t, 3H, CH_3_), 1.30 (m, 6H, 3CH_2_), 1.56 (m, 2H, CH_2_), 3.30 (t, 2H, CH_2_NH), 7.59 (m, 2H, ArH), 7.97 (m, 4H, ArH), 8.44 (s, 1H, ArH), 8.61 (t, 1H, NH). ^13^C NMR (100 MHz, DMSO-d_6_) *δ* (ppm): 13.93 (CH_3_), 22.08 (CH_2_), 26.22 (CH_2_), 29.13 (CH_2_), 31.05 (CH_2_NH), 124.19 (Ar), 126.05 (Ar), 126.65 (Ar), 127.26 (Ar), 127.44 (Ar), 127.58 (Ar), 127.77 (Ar), 132.09 (Ar), 132.16 (Ar), 134.03 (Ar), 166.08 (C=O).

#### 4.1.2 *N*-heptyl-2-naphthamide (naphthamide 7C)

A white precipitate was obtained in 75% yield. ^1^H NMR (400 MHz, DMSO-d_6_) *δ* (ppm): 0.86 (t, 3H, CH_3_), 1.26 (m, 4H, 2CH_2_), 1.31 (m, 2H, 2CH_2_), 1.57 (m, 2H, CH_2_), 3.31 (t, 2H, CH_2_NH), 7.60 (m, 2H, ArH), 7.98 (m, 4H, ArH), 8.45 (s, 1H, ArH), 8.62 (t, 1H, NH). ^13^C NMR (100 MHz, DMSO-d_6_) *δ* (ppm): 13.95 (CH_3_), 22.08 (CH_2_), 26.51 (CH_2_), 28.49 (CH_2_), 29.16 (CH_2_), 31.26 (CH_2_NH), 124.19 (Ar), 126.05 (Ar), 127.26 (Ar), 127.44 (Ar), 127.58 (Ar), 127.77 (Ar), 128.78 (Ar), 132.09 (Ar), 132.16 (Ar), 134.03 (Ar), 166.08 (C=O).

#### 4.1.3 *N*-octyl-2-naphthamide (naphthamide 8C)

A white precipitate was obtained in 78% yield. ^1^H NMR (400 MHz, DMSO-d_6_) *δ* (ppm): 0.85 (t, 3H, CH_3_), 1.25 (m, 6H, 3CH_2_), 1.31 (m, 4H, 2CH_2_), 1.57 (m, 2H, CH_2_), 3.30 (t, 2H, CH_2_NH), 7.60 (m, 2H, ArH), 7.97 (m, 4H, ArH), 8.44 (s, 1H, ArH), 8.61 (t, 1H, NH). ^13^C NMR (100 MHz, DMSO-d_6_) *δ* (ppm): 13.95 (CH_3_), 22.10 (CH_2_), 26.55 (CH_2_), 28.68 (CH_2_), 28.79 (CH_2_), 29.15 (CH_2_), 31.27 (CH_2_NH), 124.19 (Ar), 126.65 (Ar), 127.26 (Ar), 127.44 (Ar), 127.58 (Ar), 127.77 (Ar), 128.78 (Ar), 132.09 (Ar), 132.16 (Ar), 134.03 (Ar), 166.08 (C=O).

#### 4.1.4 *N*-nonyl-2-naphthamide (naphthamide 9C)

A white precipitate was obtained in 82% yield. ^1^H NMR (400 MHz, DMSO-d_6_) *δ* (ppm): 0.85 (t, 3H, CH_3_), 1.24 (m, 12H, 6CH_2_), 1.56 (m, 2H, CH_2_), 3.30 (t, 2H, CH_2_NH), 7.60 (m, 2H, ArH), 7.97 (m, 4H, ArH), 8.45 (s, 1H, ArH), 8.61 (t, 1H, NH).

### 4.2. General procedures for synthesis of 4-(dimethylamino)-*N*-alkylbenzamides (benzamide 6C–9C)

A mixture of 4-(dimethylamino) benzoic acid (1 g, 6.05 mmol) and primary aliphatic amines (1 equiv., 6.05 mmol) were dissolved in DMF (10 ml) then DCC (1.2 equiv., 1.49 g, 7.26 mmol) and 1-hydroxybenzotriazole (1.11 equiv., 0.981 g, 6.71 mmol) were added, and the reaction mixture was stirred for 12 h at room temperature. A white precipitate formed, which was filtered off and the remaining solution was poured onto ice-water mixture, the formed precipitate was collected, dried, and recrystallized from diluted ethanol.

#### 4.2.1 4-(Dimethylamino)-*N*-hexyl benzamide (benzamide 6C)

A white precipitate was collected in 82% yield. ^1^H NMR (400 MHz, DMSO-d_6_) *δ* (ppm): 0.86 (t, 3H, CH_3_), 1.25 (m, 6H, 3CH_2_), 1.27 (m, 6H, 3CH_2_), 1.49 (m, 2H, CH_2_), 2.95 (s, 6H, 2CH_3_–N), 3.22 (t, 2H, CH_2_NH), 6.67 (d, 2H, ArH), 7.70 (d, 2H, ArH), 8.06 (t, 1H, NH). ^13^C NMR (100 MHz, DMSO-d_6_) *δ* (ppm): 14.16 (CH_3_), 22.30 (CH_2_), 26.44 (CH_2_), 29.58 (CH_2_), 31.30 (CH_2NH_), 40.15 (CH_3N_), 110.95 (Ar), 121.71 (Ar), 128.63 (Ar), 152.15 (Ar), 166.16 (C=O).

#### 4.2.2 4-(Dimethylamino)-*N*-heptyl benzamide (benzamide 7C)

A white precipitate was collected in 75% yield. ^1^H NMR (400 MHz, DMSO-d_6_) *δ* (ppm): 0.86 (t, 3H, CH_3_), 1.27–1.71 (m, 10H, 5CH_2_), 1.27 (m, 6H, 3CH_2_), 1.49 (m, 2H, CH_2_), 2.95 (s, 6H, 2CH_3_–N), 3.21 (t, 2H, CH_2_NH), 6.67 (d, 2H, ArH), 7.70 (d, 2H, ArH), 8.07 (t, 1H, NH). ^13^C NMR (100 MHz, DMSO-d_6_) *δ* (ppm): 14.13 (CH_3_), 22.29 (CH_2_), 26.43 (CH_2_), 29.57 (CH_2_), 31.29 (CH_2NH_), 40.15 (CH_3N_), 110.95 (Ar), 121.70 (Ar), 128.62 (Ar), 152.16 (Ar), 166.14 (C=O).

#### 4.2.3 4-(Dimethylamino)-*N*-octylbenzamide (benzamide 8C)

A white precipitate was collected in 85% yield. ^1^H NMR (400 MHz, DMSO-d6) *δ* (ppm): 0.85 (t, 3H, CH_3_), 1.26–1.49 (m, 12H, 6CH_2_), 2.95 (s, 6H, 2CH_3_–N), 3.19 (t, 2H, CH_2_NH), 6.67 (d, 2H, ArH), 7.70 (d, 2H, ArH), 8.06 (t, 1H, NH). ^13^C NMR (100 MHz, DMSO-d_6_) *δ* (ppm): 14.16 (CH_3_), 22.31 (CH_2_), 26.77 (CH_2_), 28.91 (CH_2_), 29.02 (CH_2_), 29.60 (CH_2_), 31.48 (CH_2NH_), 40.15 (CH_3N_), 110.95 (Ar), 121.70 (Ar), 128.62 (Ar), 152.16 (Ar), 166.14 (C=O).

#### 4.2.4 4-(Dimethylamino)-*N*-nonylbenzamide (benzamide 9C)

A white precipitate was collected in 80% yield. ^1^H NMR (400 MHz, DMSO-d_6_) *δ* (ppm): 0.85 (t, 3H, CH_3_), 1.24–1.71 (m, 14H, 7CH_2_), 2.95 (s, 6H, 2CH_3_–N), 3.19 (t, 2H, CH_2_NH), 6.67 (d, 2H, ArH), 7.70 (d, 2H, ArH), 8.06 (t, 1H, NH). ^13^C NMR (100 MHz, DMSO-d_6_) *δ* (ppm): 14.16 (CH_3_), 22.31 (CH_2_), 26.75 (CH_2_), 28.89 (CH_2_), 29.05 (CH_2_), 29.20 (CH_2_), 29.58 (CH_2_), 31.50 (CH_2_NH), 40.15 (CH_3N_), 110.95 (Ar), 121.69 (Ar), 128.62 (Ar), 152.15 (Ar), 166.13 (C=O).

## Data availability

Mostafa Sayed and Mostafa Ahmed, the corresponding authors and all the coauthors, confirm that all the data associated with this article is available in the main article and ESI.†

## Conflicts of interest

The authors state that there are no conflicts to declare.

## Supplementary Material

RA-015-D5RA00978B-s001
