# Supplementary material for: Molecular design and performance of emissive amide-containing compounds as corrosion inhibitors: synthesis, electrochemical evaluation, DFT calculations and molecular dynamics simulations
Source: RSC Adv. 2025 May 9;15(19):15384–96. doi: 10.1039/d5ra00978b (PMC12063569; doi:10.1039/d5ra00978b)
Supplement: RA-015-D5RA00978B-s001 [file RA-015-D5RA00978B-s001.pdf]

Supplementary Information (SI) for RSC Advances.
This journal is © The Royal Society of Chemistry 2025

Molecular Design and Performance of Emissive Amide-Containing Compounds as Corrosion Inhibitors: Synthesis, Electrochemical Evaluation, DFT Calculations and Molecular Dynamic Simulations

Abdelreheem A. Saddik[1,2,#], Mostafa Sayd[3,#,*], Ahmed A.K. Mohammed[1], Mohamed Abdel-Hakim[4], Mostafa Ahmed[3,*]

[1] Department of Chemistry, Faculty of Science, Assiut University, Assiut 71516, Egypt

[2] Innovation Incubation Center, National Tsing Hua University, Hsinchu, 300, Taiwan

[3] Chemistry Department, Faculty of Science, New Valley University, El-Kharja 72511, Egypt

[4] Department of Chemistry, Faculty of Science, Al-Azhar University, Assiut 71524, Egypt

*Address correspondence to these authors at the Chemistry Department, Faculty of Science, New Valley University, El-Kharja 72511, Egypt; E-mail: mostafaali@mail.ustc.edu.cn and mostafasayed@sci.nvu.edu.eg (Mostafa Sayed), E-mail: drmostafa@scinv.au.edu.eg (Mostafa Ahmed)

#These authors contributed equally to this work (Abdelreheem A. Saddik and Mostafa Sayed)

1. Electrochemical Study

1.2. Instrumentations

Using the 352/252 model corrosion measuring technique, an EG&G potentiostat/galvanostat model 273A, and IBM software, electrochemical experiments open circuit potential and (linear, Tafel plots polarizations) tests are carried out (Al-Azhar University - Assuit).

1.3. Preparing studied surface and tested media

The composition of the mild steel (MS) specimen is (wt.%) Fe 98%. For the electrochemical studies, divided the MS specimens into $1\times1\times1 cm^3$. The surfaces of all tested samples were polished with different grades of emery polishing papers such as 1200 and 1400, then degreased with acetone, and finally dried. The corrosive solutions were prepared by analytical grade 97 % H_2SO_4 (Sigma-Aldrich Laborchemikalien, German) with dilution by bi-distilled water.

1.4 Preparation of corrosive and inhibitor solutions

Amide solutions from compounds **naphthamide 6C-9C** and **benzamide 6C-9C** as inhibitors that were prepared by weighing 0.02g of the tested inhibitors in ethyl alcohol as solvent to obtain 200 ppm of each inhibitor needed to complete the experiment, Mild steel (working electrode) pickling for 15 minutes in prepared inhibitors solution then applying the electrochemical experiments in 100 cm^3 of 1.0 M H_2SO_4.

1.5. Electrochemical Techniques

The potentiodynamic method used in this study includes open circuit potential (OCP) for immersion MS electrodes potential (E_{im}) in the blank solution (without) and with inhibitors concentrations to obtain a steady-state potential (Es.s) which is nearby corrosion potential ($E_{corr} \approx E_{ocp}$). Potentiodynamic polarization (PP), which records parameters such as corrosion potential (E_{corr} mV), corrosion current density (I_{corr} $\mu A/cm^2$), corrosion rate (C.R millimeter per year mpy), inhibition efficiency percentage (IE%), and surface coverage (θ =IE%/100). OCP is performed using reference electrode as (SEC) and working (MS), but in PP experiments, add a counter electrode (Pt wire) with the obvious OCP electrodes. OCP and PP curves performed by EG & G potentiostat/galvanostat instrument model 273A. TF was scanned at ±250 mV vs. $E_{corr\approx ocp}$ with the rate of scan 0.3 mV/Sec. CR and IE% for the tested inhibitors were

calculated from I_{corr} mathematically according to the following Eqs. 3 and 4 respectively.[1]

$$CR = \frac{0.13 \times Icorr \times Eq.Wt}{\rho \times A} \text{............}(3)$$

Where CR is the (corrosion rate mpy), $\mathbf{I_{corr}}$ is the (corrosion current density μA/cm^2), which records the current value at which the corrosion process takes place, Eq. Wt. is the equivalent weight of the metal (gm/eq) equal to 55.8 Atomic mass, A is the area (cm^2) immersed in tested solutions, ρ is the (density gm/cm^3) equal to 7.874 g/cm^3, and 0.13 is the metric and time conversion factor.

$$IE\% = \frac{CR - CR1}{CR} \times 100 \text{ ....}(4)$$

The corrosion rates without and with inhibitors, respectively, are CR and CR1.

2. Photophysical Study

UV-Vis absorption spectra were measured with a spectropolarimeter (HP8453 UV-Visible spectrophotometer, cell length 1 mm, 25°C), and Fluorescence emission spectra

3.1 Quantum-chemical calculations

We performed density functional theory (DFT) calculations on all eight amides to reveal their structure and gain insights into their properties. The ground state geometry of each molecule was optimized using DFT with the B3LYP functional with the D3BJ correction and the basis set 6-31G(d). This dispersion correction is necessary to account for long-range and noncovalent interactions, such as van der Waals forces. Several conformers were considered for the ground state geometry, and the one with lowest energy was selected. These geometries were confirmed as true minima by the absence of imaginary frequencies. All geometry optimizations were performed using the Gaussian 16 software.

3.2 Molecular dynamics (MD) simulations

We performed molecular dynamics simulations in a box with regular boundary conditions. An Fe crystal was impressed and split along the (110) plane. To ensure enough metal surface is available for interaction with the inhibitor, a supercell with the dimensions a=19.9Å, b=29.8Å, and c=20.0Å was created. The MD simulations were performed using the forcite module implemented in Materials Studio software. The inhibitor was placed on the Fe surface and the supercell was filled with water. Six

H_2SO_4 molecules were added to the simulation box to capture the effect of the corrosive ions. The system was first relaxed by an energy minimization using the generic universal forcefield (UFF). Then MD simulations were performed with the NVT ensemble (volume, and temperature, and number of particles were kept constant) with a time step of 1 fs and a simulation time of 1ns at 1 bar pressure and 303 K temperature using UFF.

4. FT-IR, ¹H, and ¹³C NMR spectra of synthesized compounds

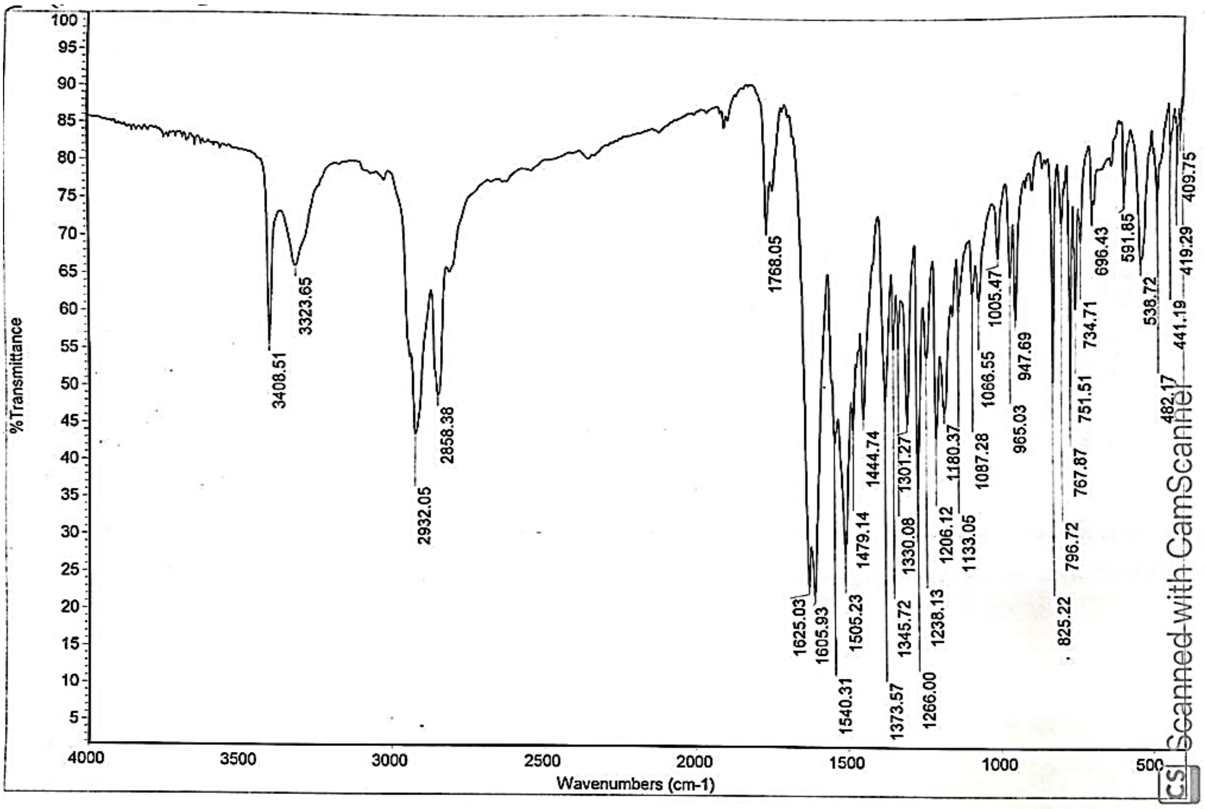

Figure S1: IR Spectrum of compound **naphthamide 6C**

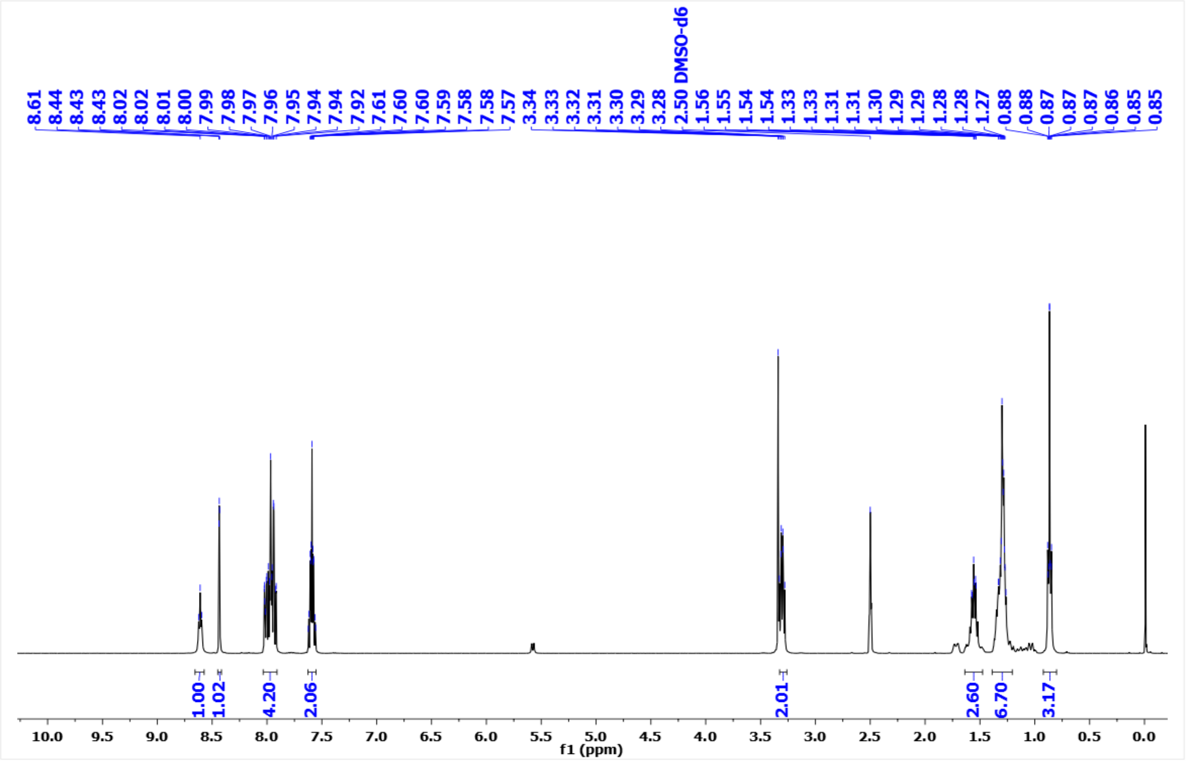

Figure S2: ¹H NMR Spectrum of compound **naphthamide 6C**

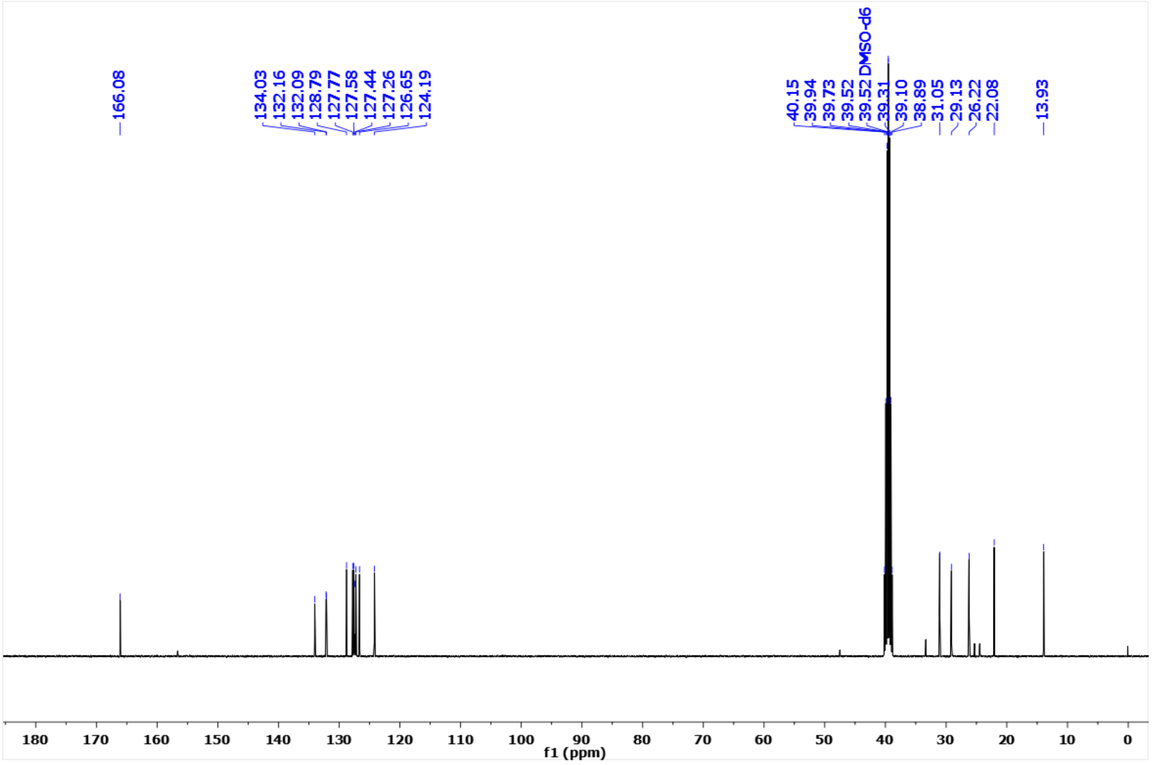

Figure S3: ^{13}C NMR Spectrum of compound **naphthamide 6C**

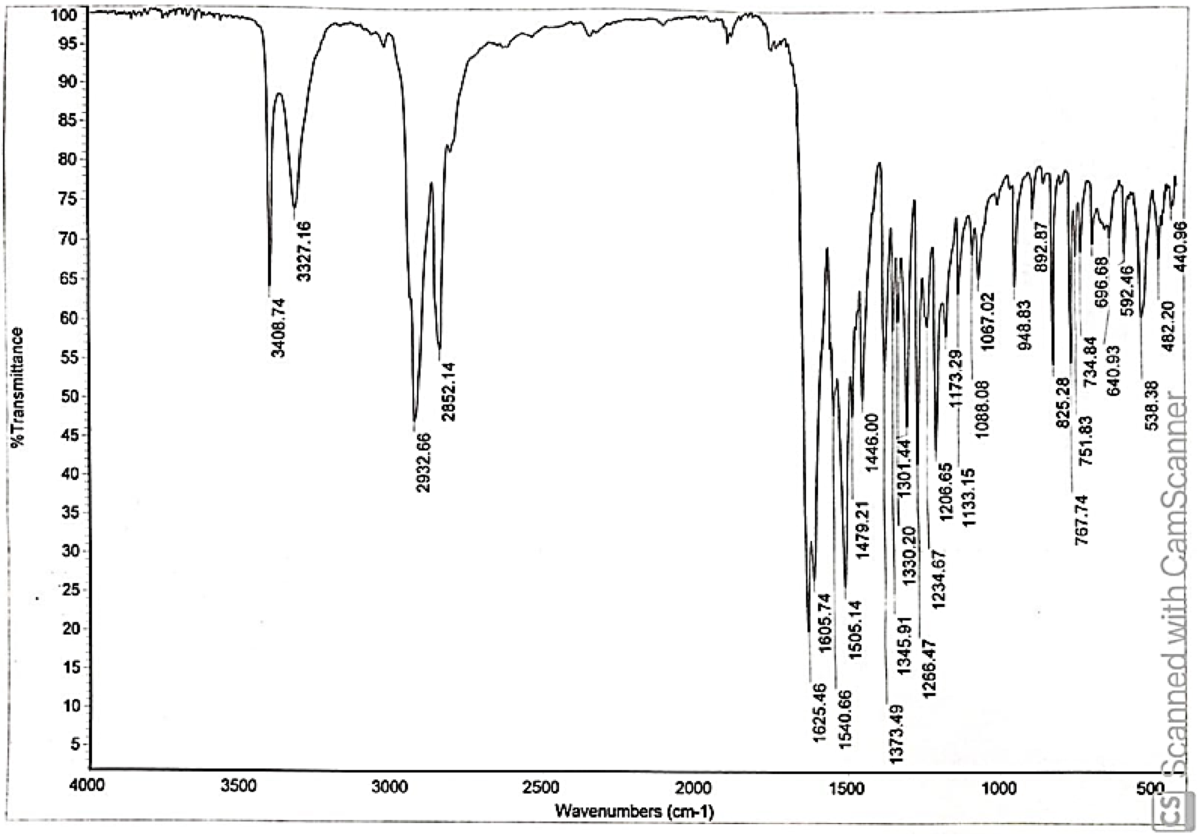

Figure S4: IR Spectrum of compound **naphthamide 7C**

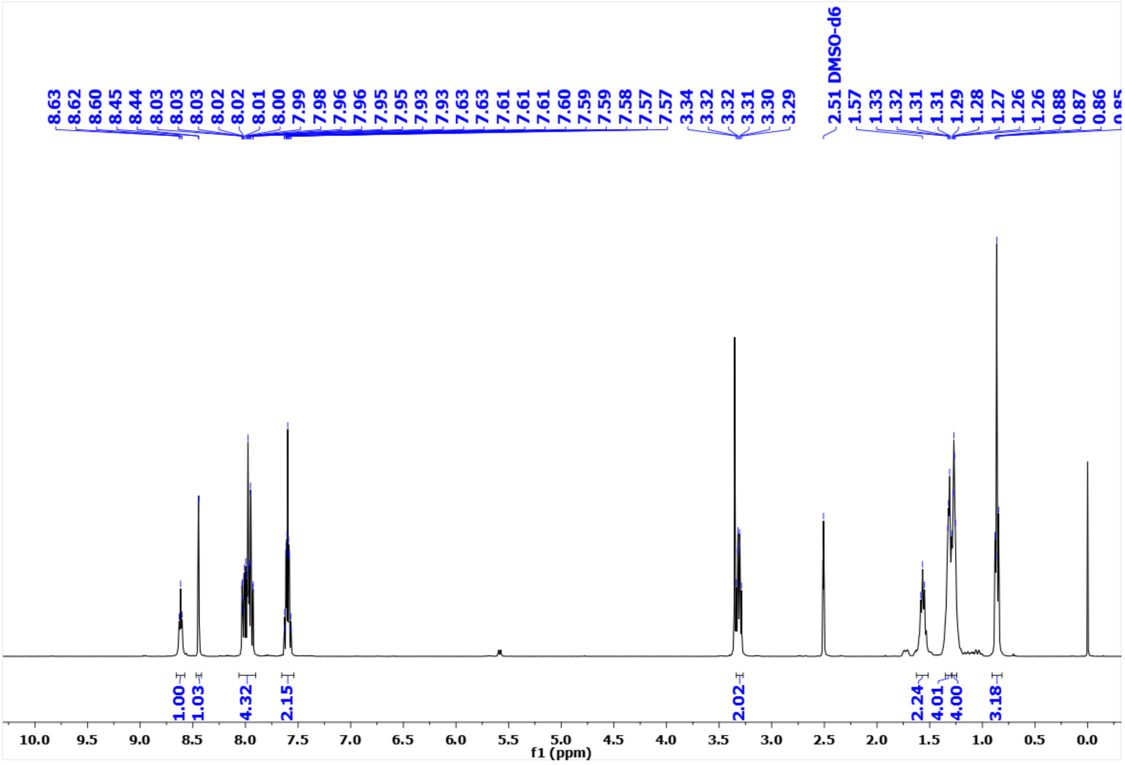

Figure S5: ^{1}H NMR Spectrum of **naphthamide 7C**

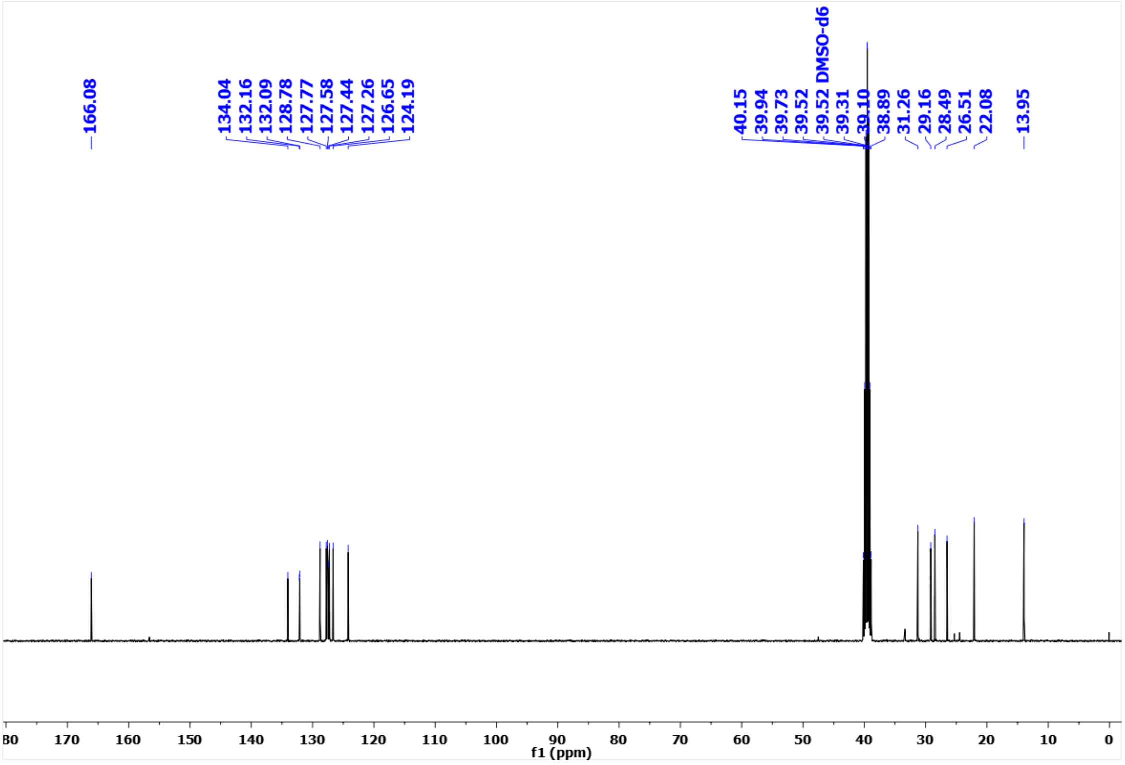

Figure S6: ^{13}C NMR Spectrum of **naphthamide 7C**

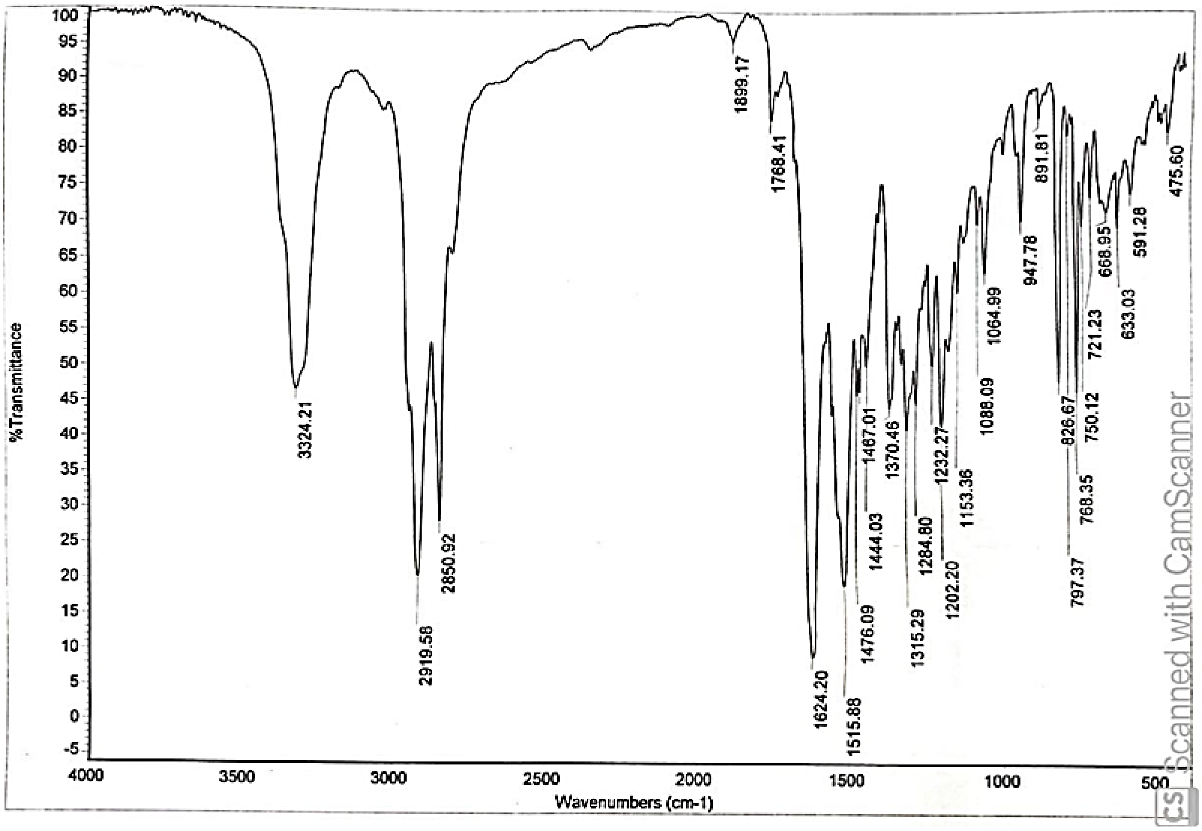

Figure S7: IR Spectrum of **naphthamide 8C**

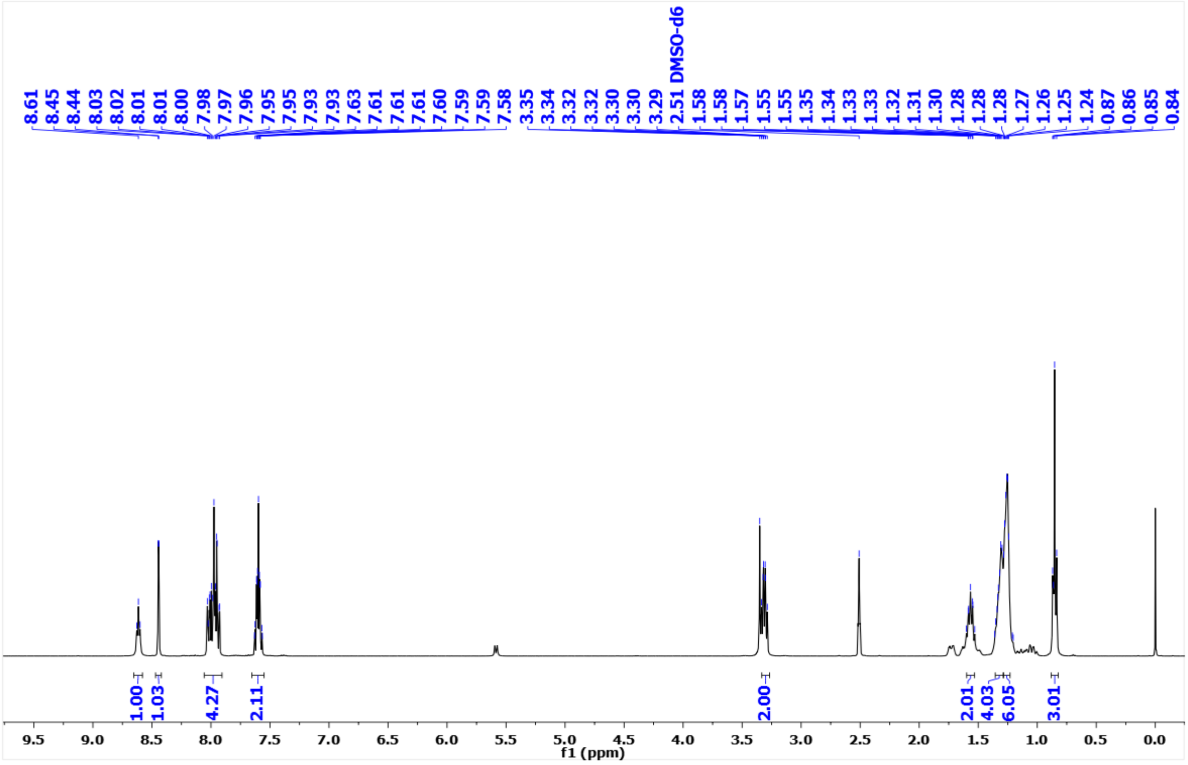

Figure S8: ^{1}H NMR Spectrum of **naphthamide 8C**

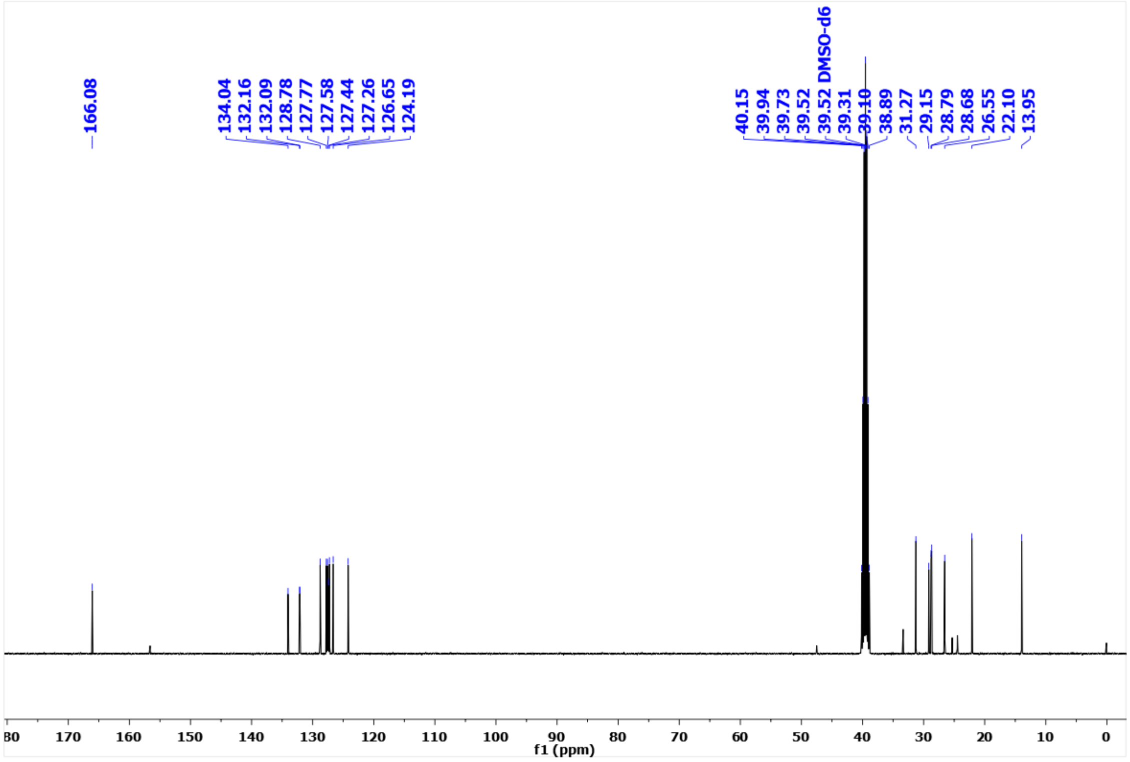

Figure S9: ^{13}C NMR Spectrum of **naphthamide 8C**

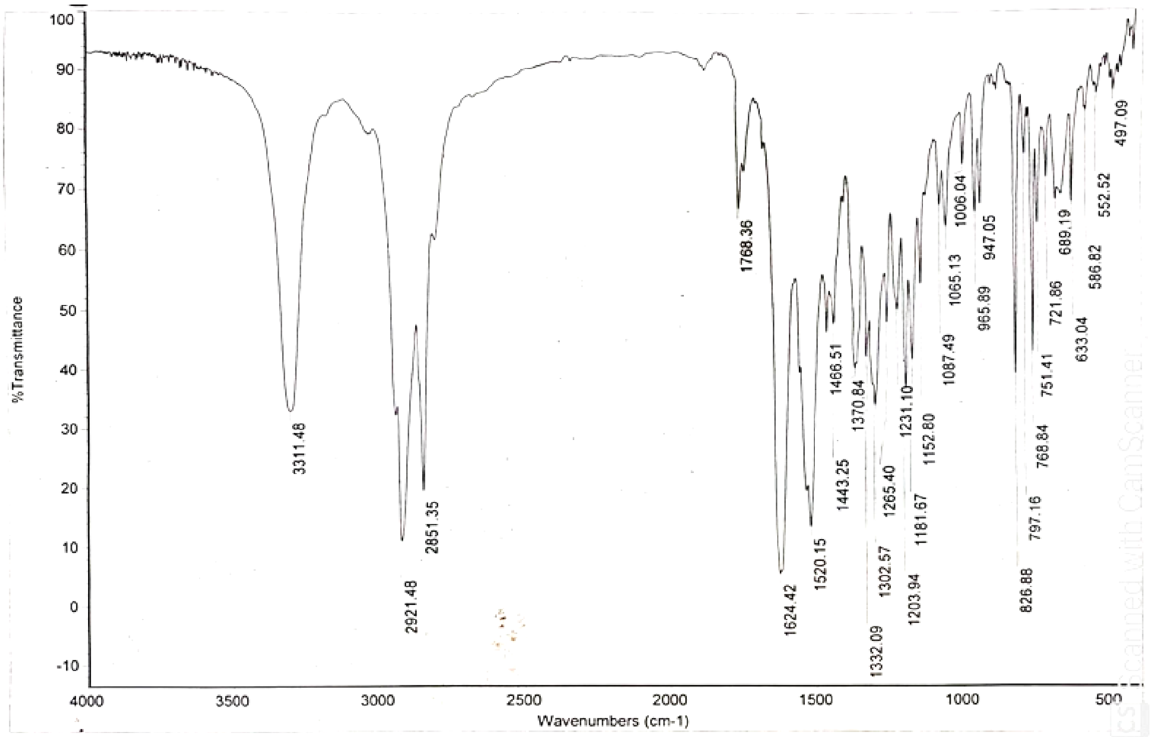

Figure S10: IR Spectrum of **naphthamide 9C**

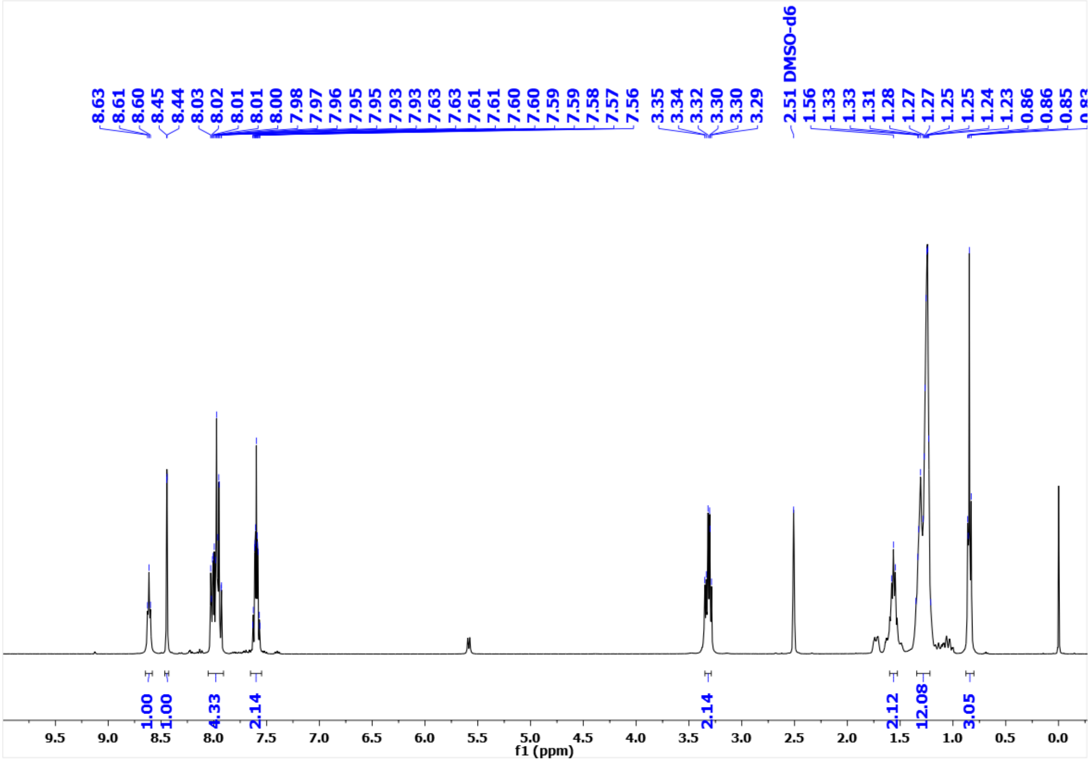

Figure S11: ^{1}H NMR Spectrum of **naphthamide 9C**

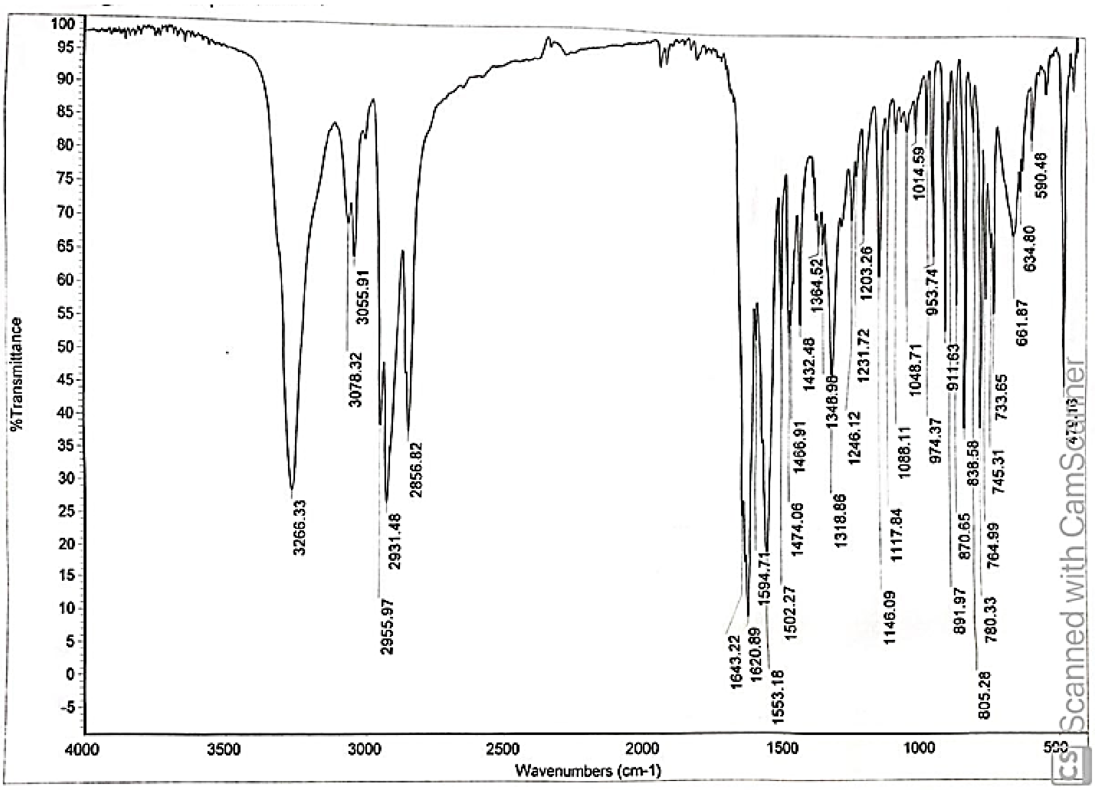

Figure S12: IR Spectrum of **benzamide 6C**

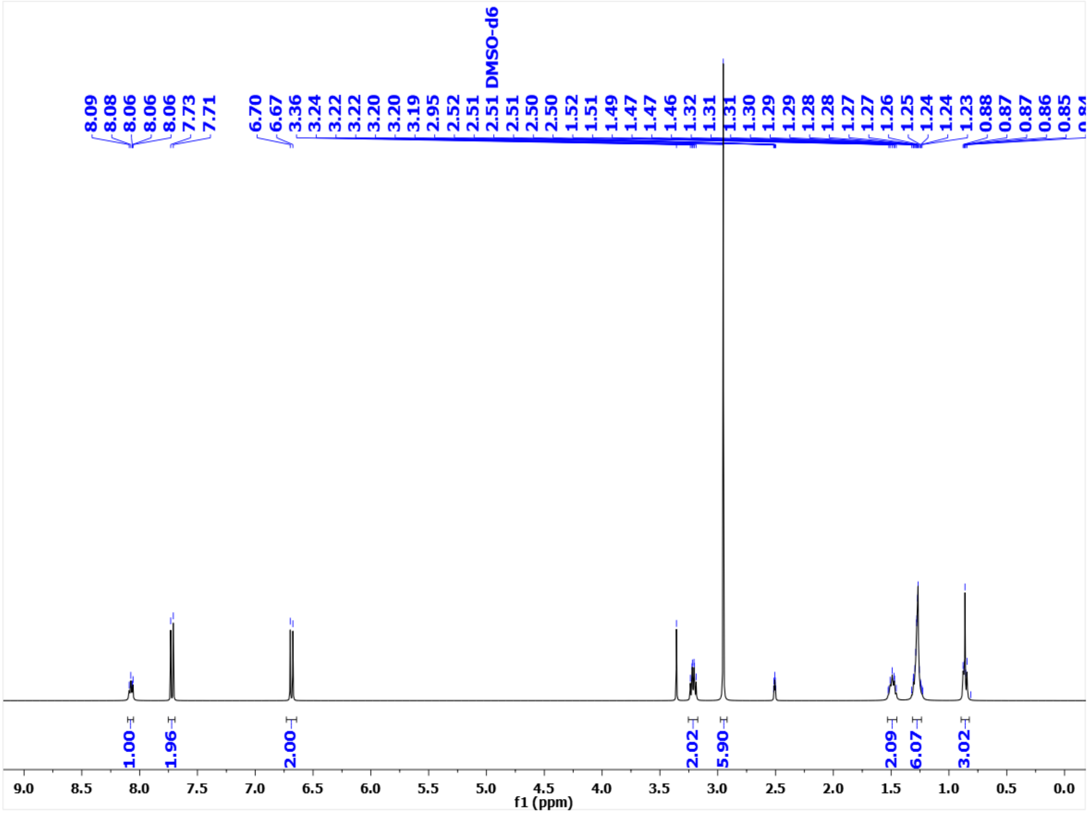

Figure S13: ^{1}H NMR Spectrum of **benzamide 6C**

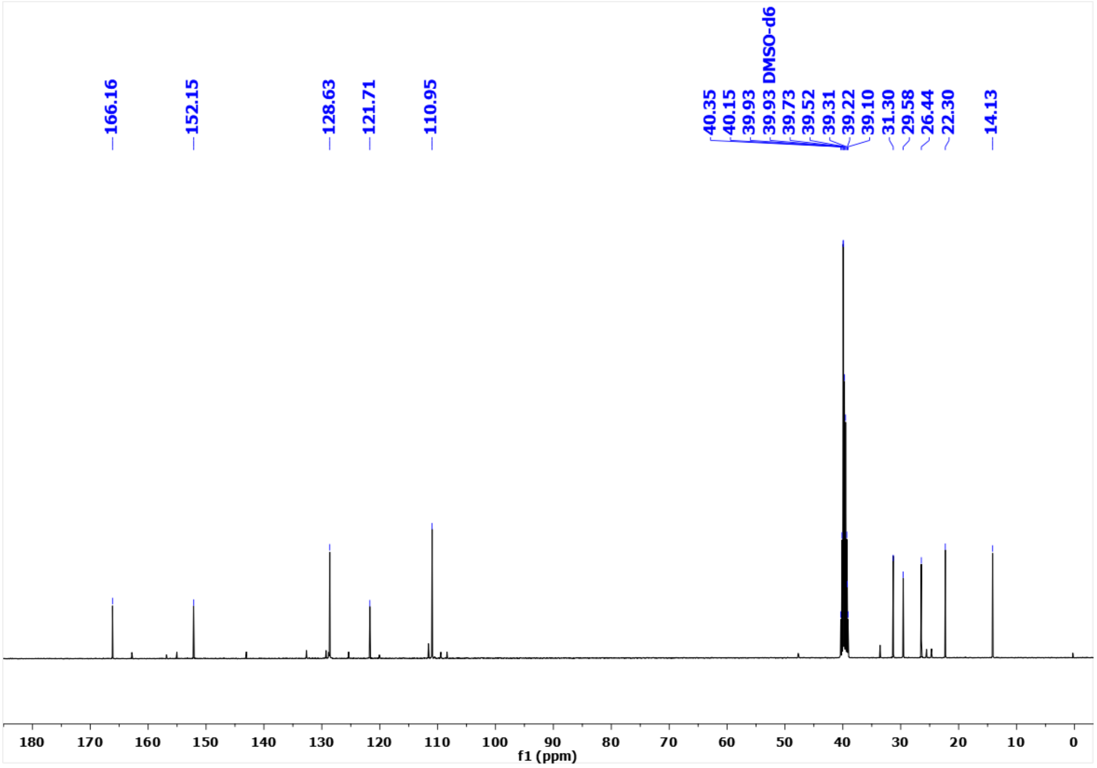

Figure S14: ^{13}C NMR Spectrum of **benzamide 6C**

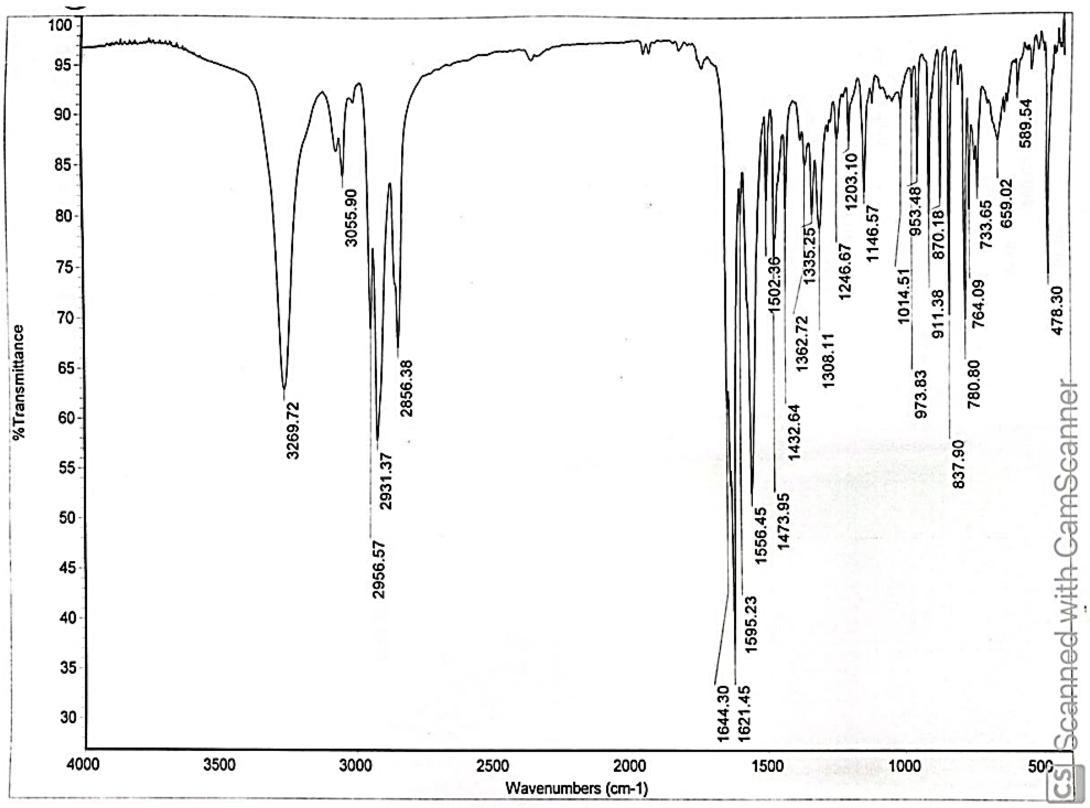

Figure S15: IR Spectrum of **benzamide 7C**

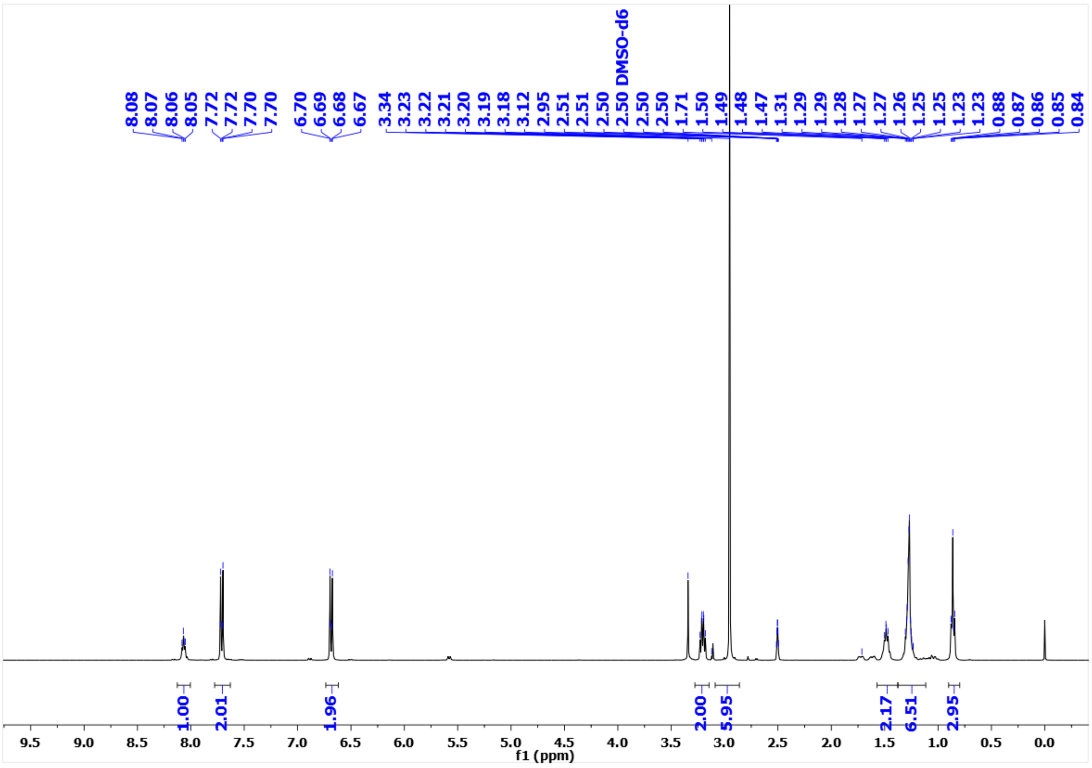

Figure S16: ^{1}H NMR Spectrum of **benzamide 7C**

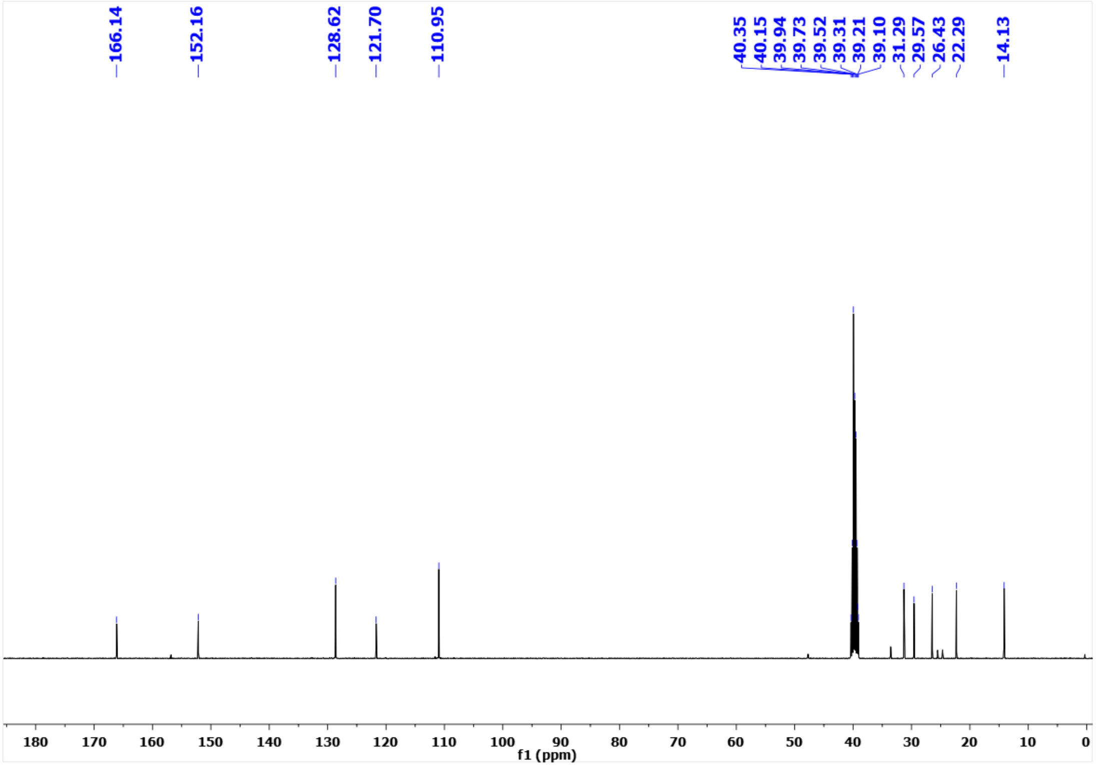

Figure S17: ^{13}C NMR Spectrum of **benzamide 7C**

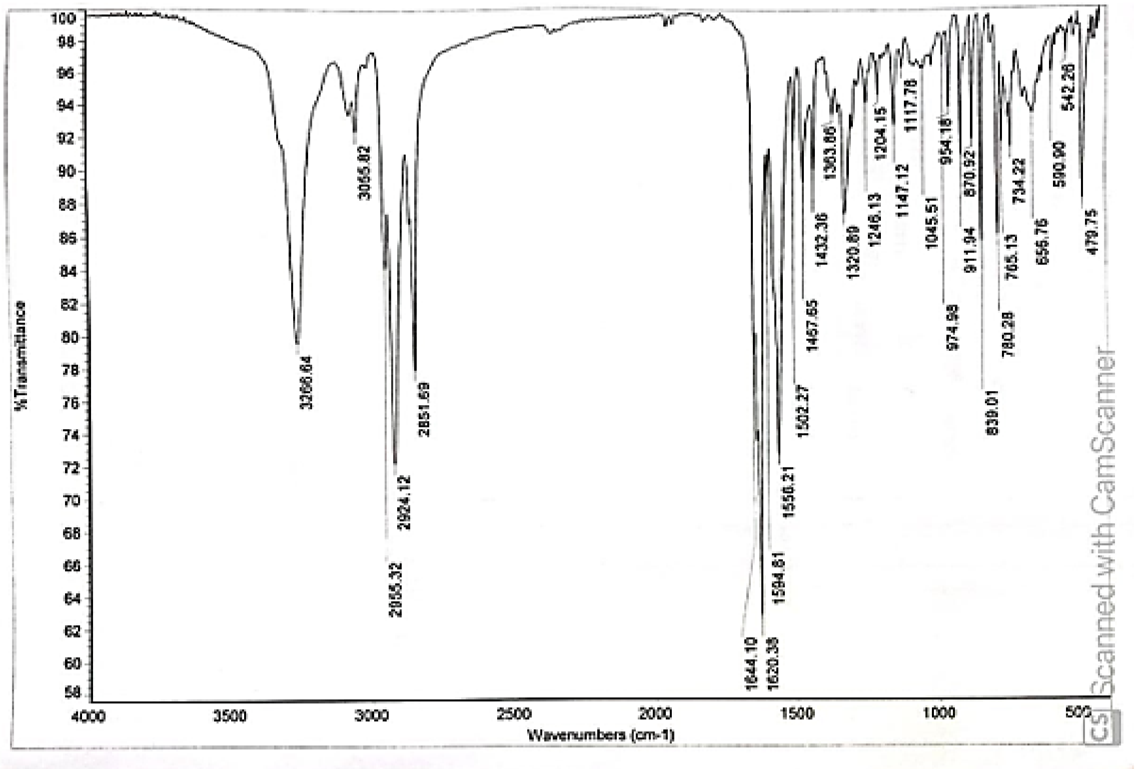

Figure S18: IR Spectrum of **benzamide 8C**

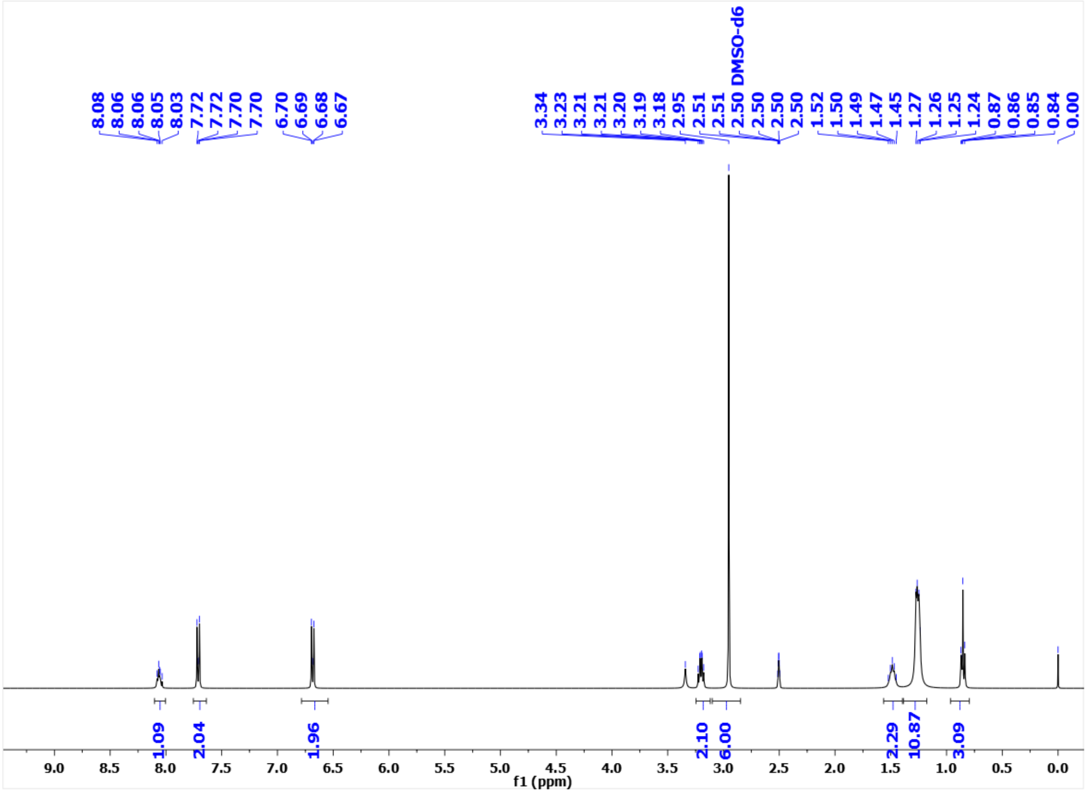

Figure S19: ^{1}H NMR Spectrum of **benzamide 8C**

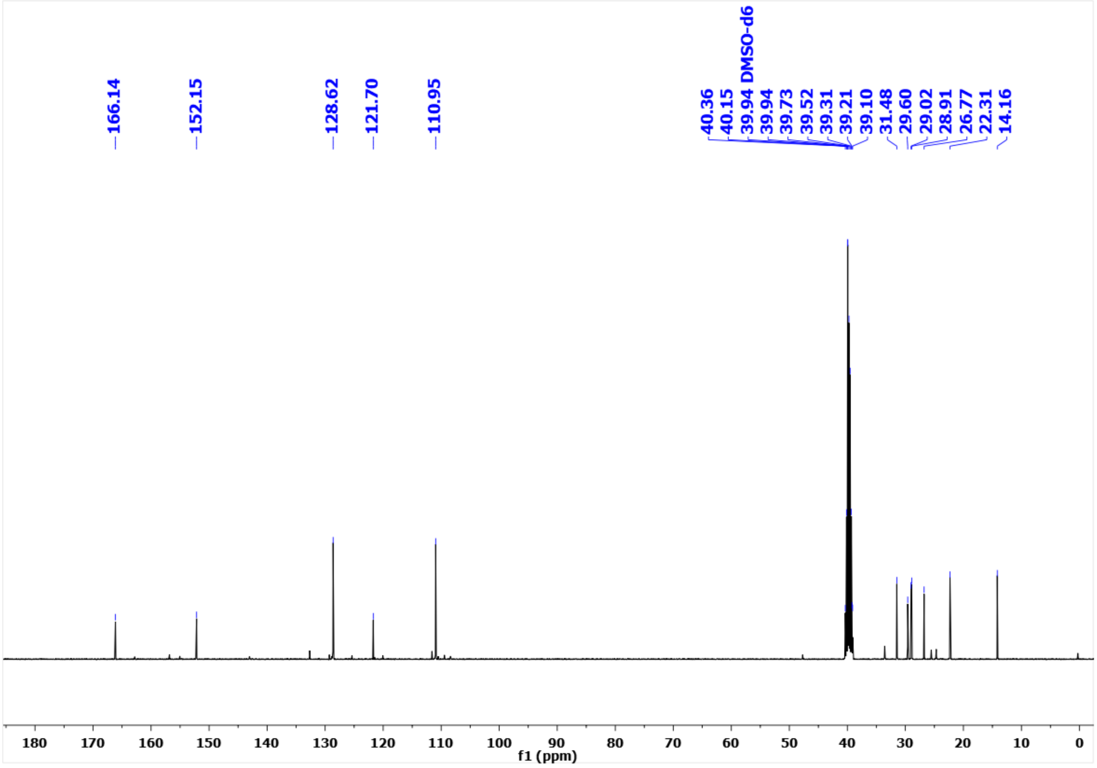

Figure S20: ^{13}C NMR Spectrum of **benzamide 8C**

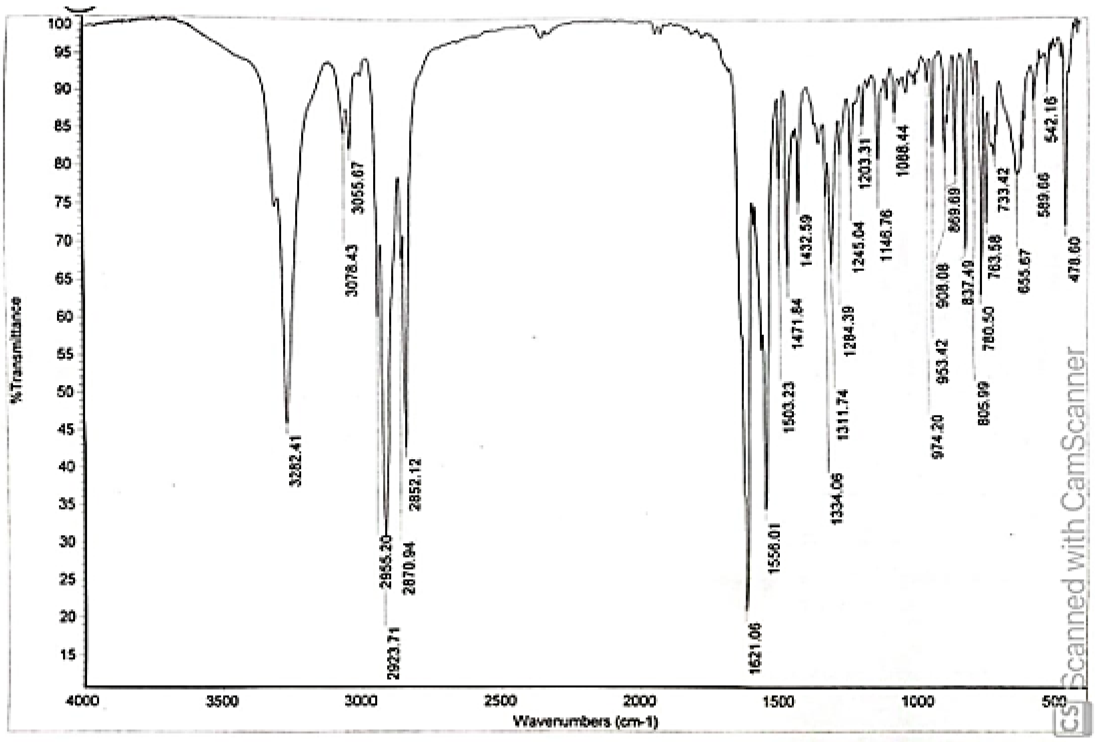

Figure S21: IR Spectrum of **benzamide 9C**

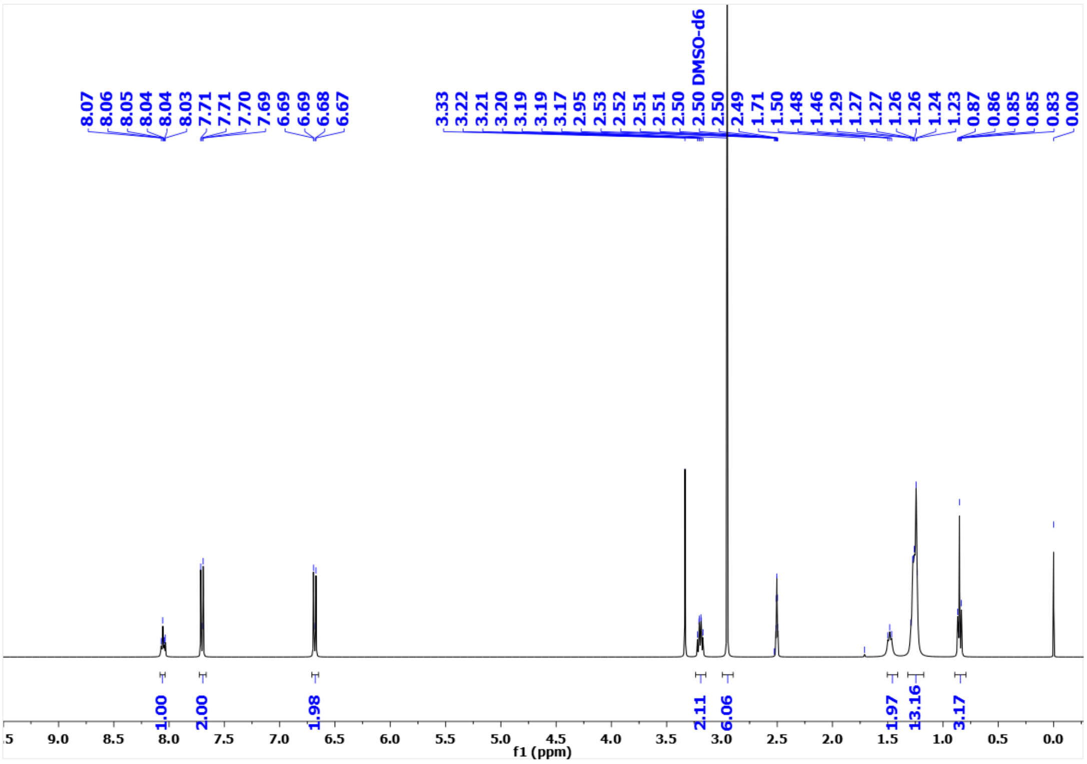

Figure S22: ^{1}H NMR Spectrum of **benzamide 9C**

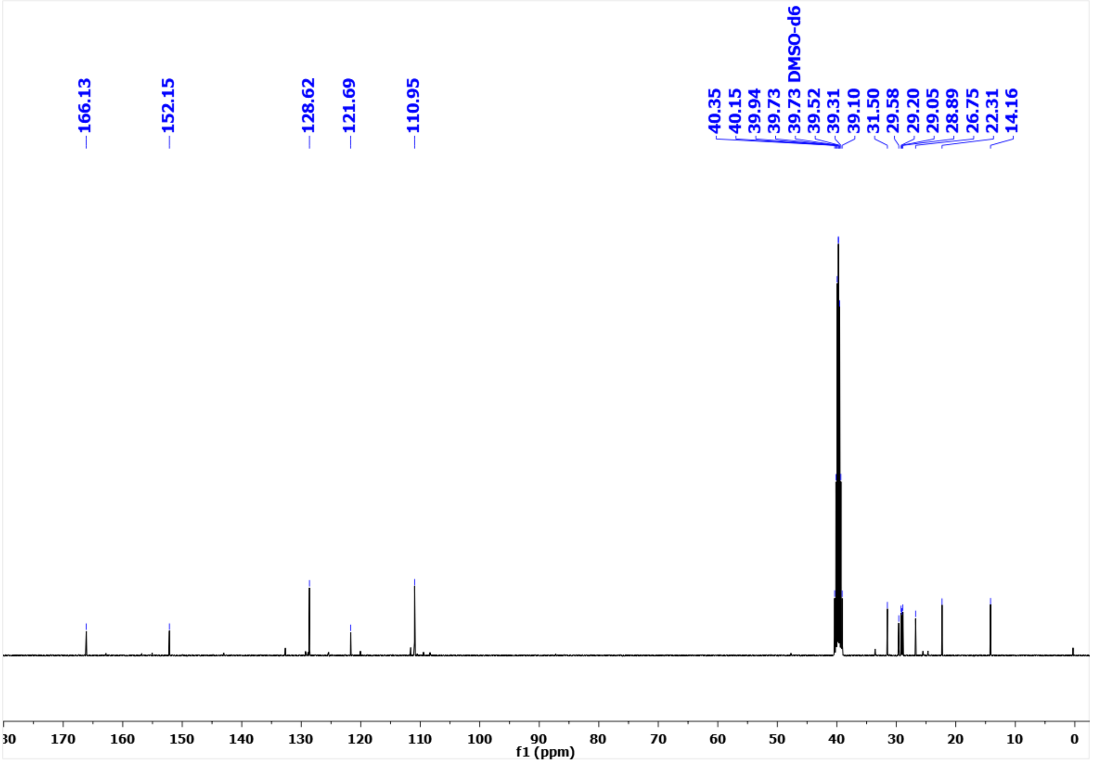

Figure S23: ^{13}C NMR Spectrum of **benzamide 9C**

5. Supplementary Figures

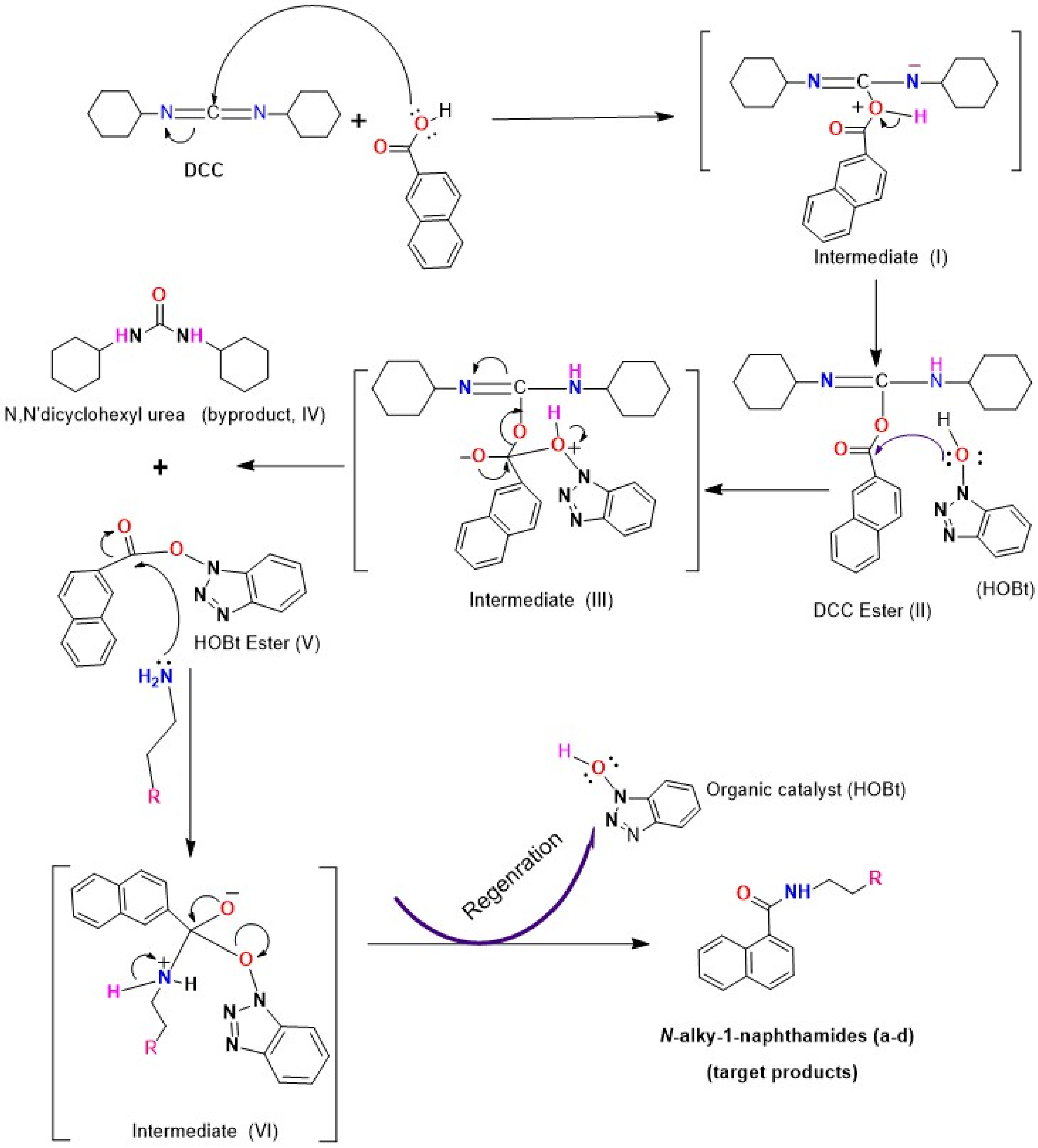

Scheme S1. Proposed Mechanism of Amide Formation.

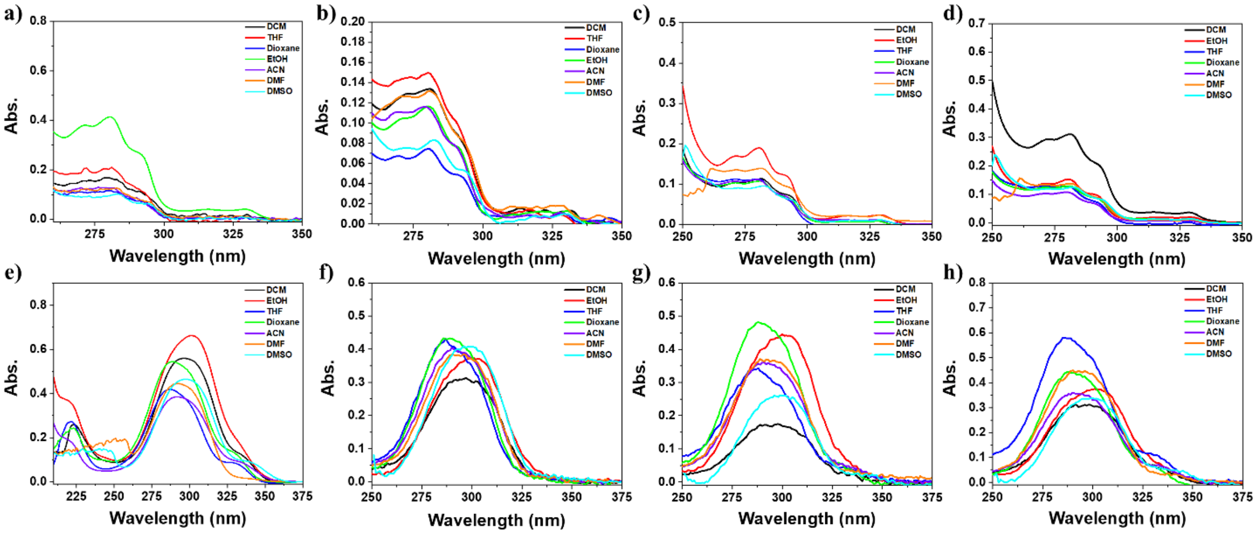

Figure S24: a-h) Absorption spectra for compounds **naphthamide 6C-9C** and **benzamide 6C-9C** solutions in different organic solvents (DCM, EtOH, THF, Dioxane, ACN, DMF, and DMSO) at 100 µM; a) compound **naphthamide 6C**, b) compound **naphthamide 7C**, c) compound **naphthamide 8C**, d) compound **naphthamide 9C**, e) compound **benzamide 6C**, f) compound **benzamide 7C**, g) compound **benzamide 8C**, h) compound **benzamide 9C**.

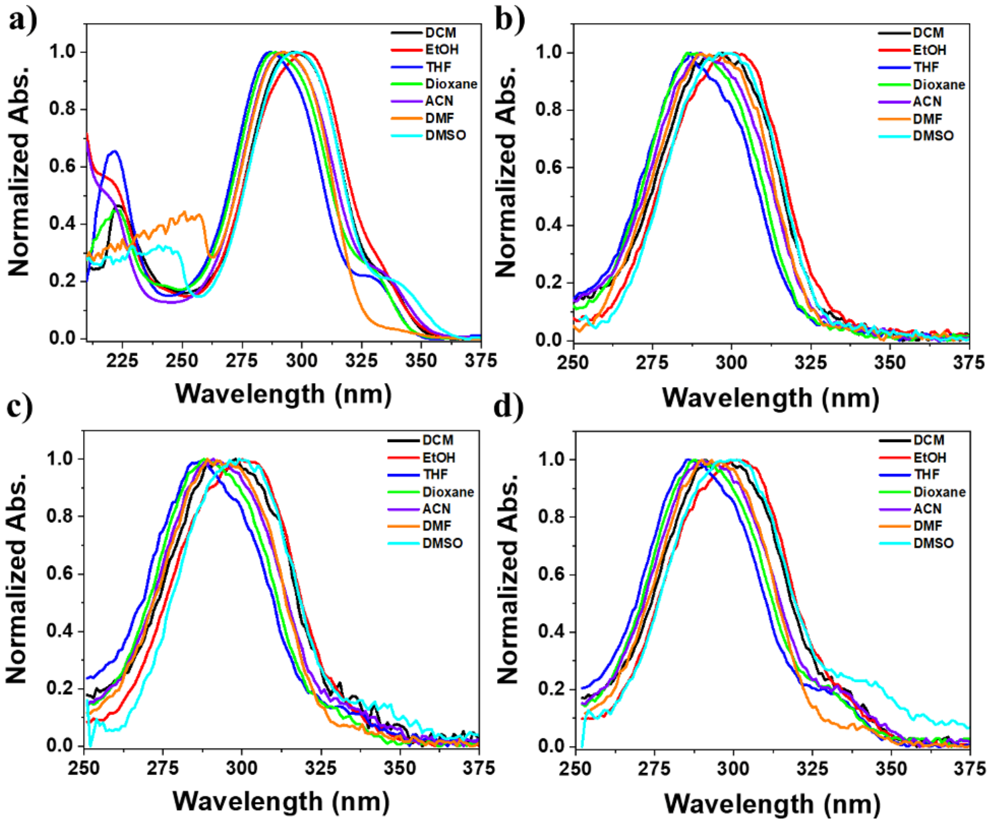

Figure S25. a-d) Normalized absorption spectra of compounds **benzamide 6C-9C** at different organic solvents (DCM, EtOH, THF, Dioxane, ACN, DMF, and DMSO) at

100 μM; a) compound **benzamide 6C**, b) compound **benzamide 7C**, c) compound **benzamide 8C**, and d) compound **benzamide 9C**.

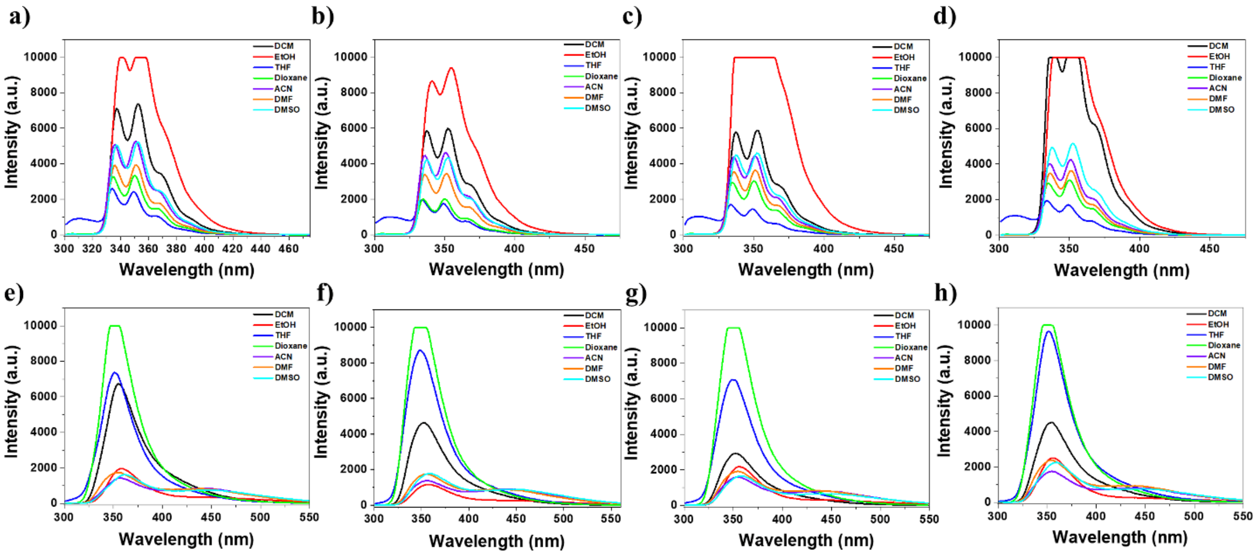

Figure S26: a-h) Emission spectra of compounds **naphthamide 6C-9C** and **benzamide 6C-9C** at different organic solvents (DCM, EtOH, THF, Dioxane, ACN, DMF, and DMSO) at 100 μM (λ_{ex} = 281 nm for compounds **naphthamide 6C-9C**, and 287 nm for compounds **benzamide 6C-9C** respectively); a) compound **naphthamide 6C**, b) compound **naphthamide 7C**, c) compound **naphthamide 8C**, d) compound **naphthamide 9C**, e) compound **benzamide 6C**, f) compound **benzamide 7C**, g) compound **benzamide 8C**, h) compound **benzamide 9C**.

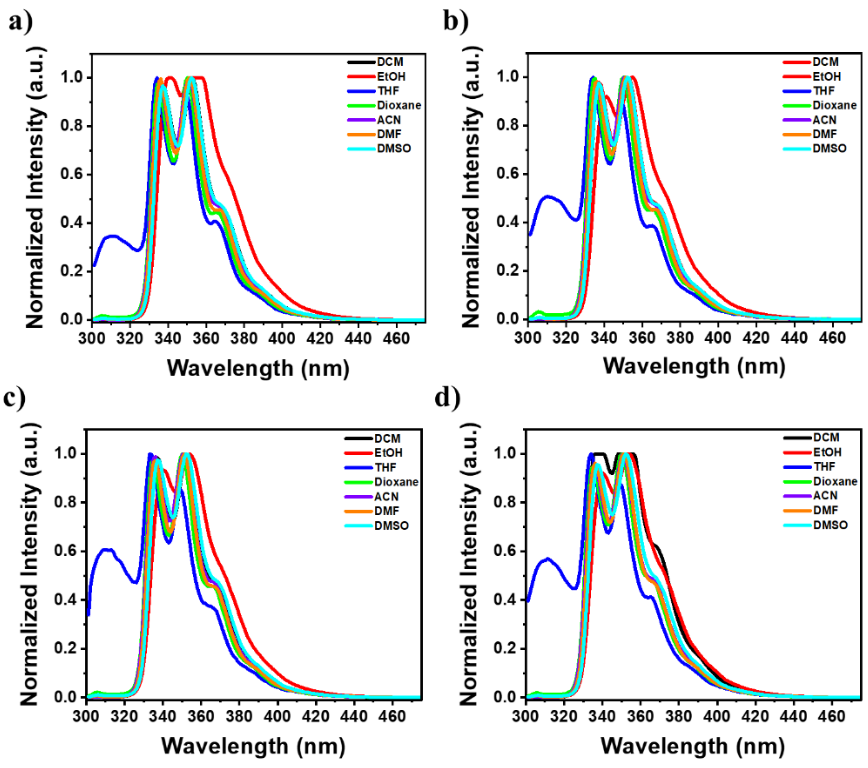

Figure S27. a-d) Normalized emission spectra of compounds **Naphthamide-6C** to **Naphthamide-9C** at different organic solvents (DCM, EtOH, THF, Dioxane, ACN, DMF, and DMSO) at 100 µM (λ_{ex} = 281 nm); a) **Naphthamide-6C**, b) **Naphthamide-7C**, c) **Naphthamide-8C**, and d) **Naphthamide-9C**.

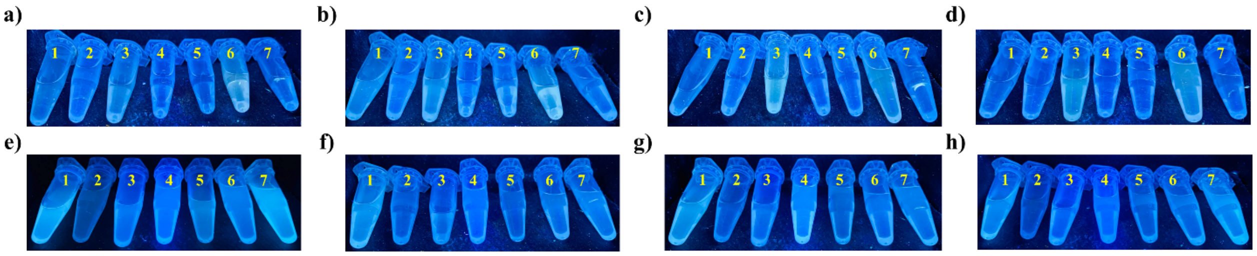

Figure S28: a-h) Optical images of the compounds **naphthamide 6C-9C** and **benzamide 6C-9C** solutions at different organic solvents under UV light (365 nm) (1; DCM, 2; EtOH, 3; THF, 4; Dioxane, 5; ACN, 6; DMF, and 8; DMSO) at 100 µM; a) compound **naphthamide 6C**, b) compound **naphthamide 7C**, c) compound **naphthamide 8C**, d) compound **naphthamide 9C**, e) compound **benzamide 6C**, f) compound **benzamide 7C**, g) compound **benzamide 8C**, h) compound **benzamide 9C**.

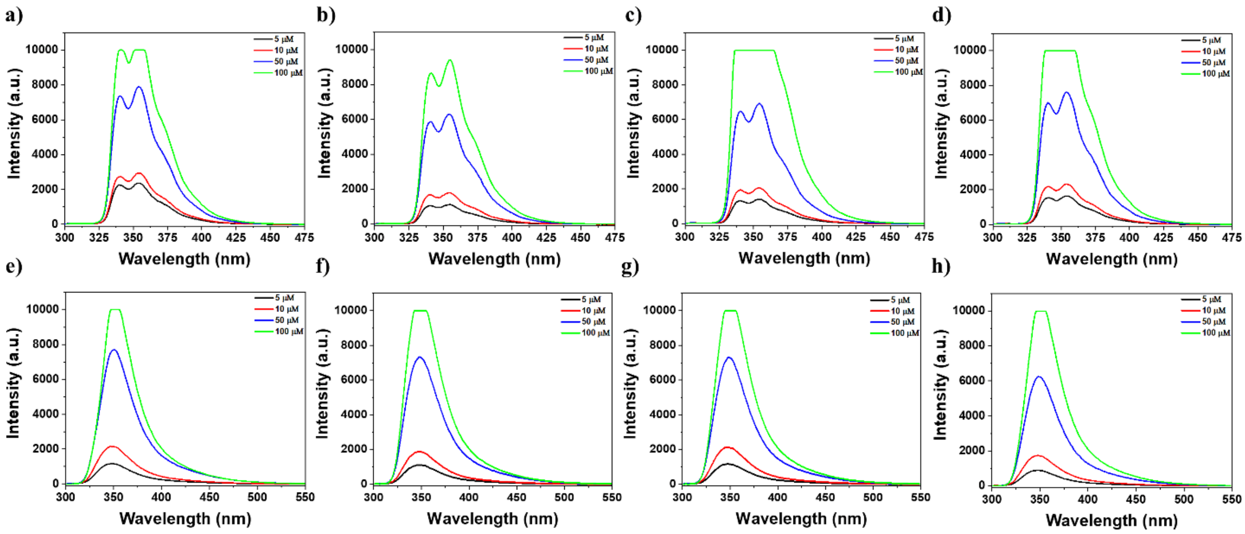

Figure S29: a-d) Emission spectra of compounds **naphthamide 6C-9C** in EtOH at different concentrations (10, 50, and 100 μM) at λ_{ex} = 281 nm; a) compound **naphthamide 6C**, b) compound **naphthamide 7C**, c) compound **naphthamide 8C**, d) compound **naphthamide 9C**. e-h) Emission spectra of compounds **benzamide 6C-9C** in dioxane at different concentrations (10, 50, and 100 μM) at λ_{ex} = 287 nm; e) compound **benzamide 6C**, f) compound **benzamide 7C**, g) compound **benzamide 8C**, h) compound **benzamide 9C**.

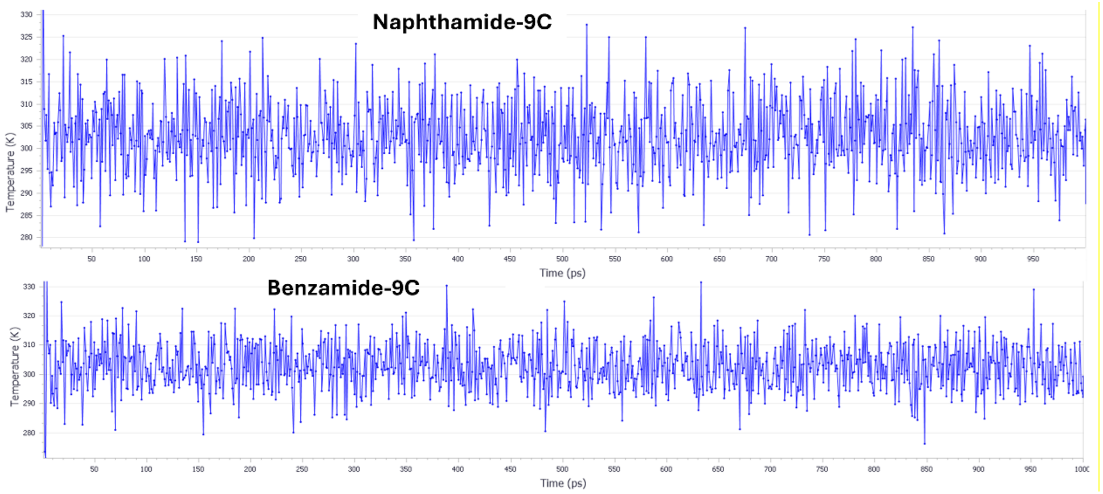

Figure S30: Temperature equilibrium curve for the MD simulations of **Naphthamide-9C** and **Benzamide-9C**.

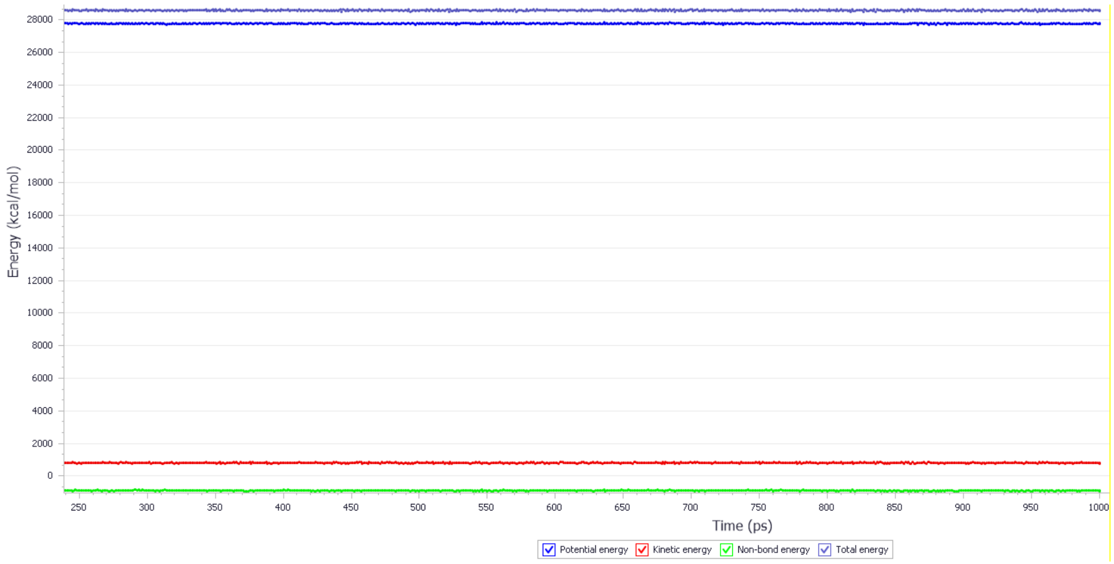

Figure S31: Energy fluctuations curve for the MD simulations of **Naphthamide-9C**.

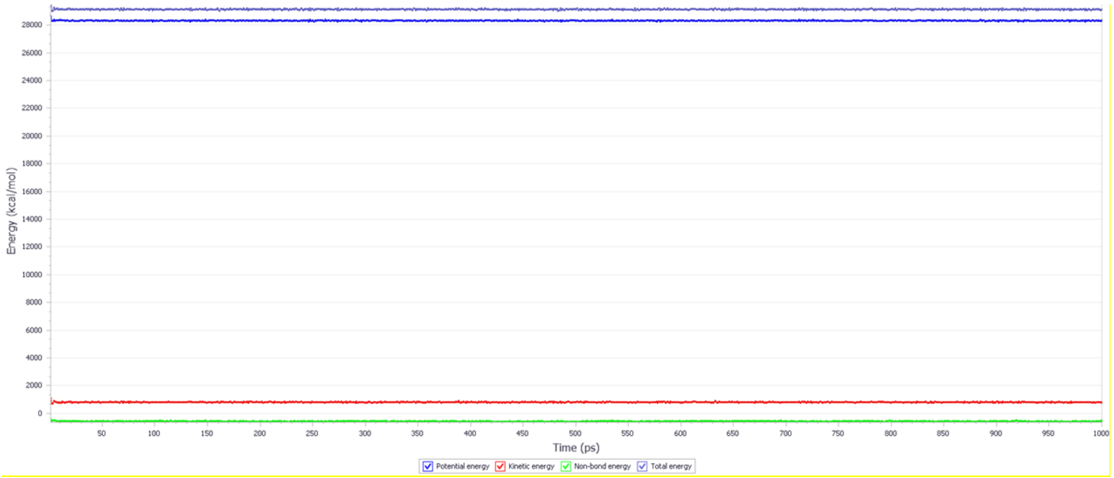

Figure S32: Energy fluctuations curve for the MD simulations of **Benzamide-9C**.

Table S1. Comparison of our amide inhibitors to verified steel corrosion inhibitors.

**Inhibitor**	**Alloy**	**Conc. of inhibitor**	**IE (%)**	**Ref.**
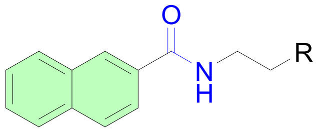	Mild Steel	200 ppm	80	This Report
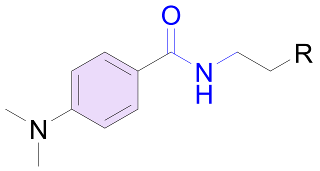	Mild Steel	200 ppm	75	This Report
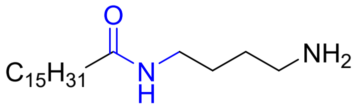	Mild Steel	0.5 mM	92	**2**
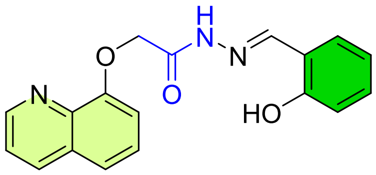	Mild Steel	0.5 mM	93	**3**
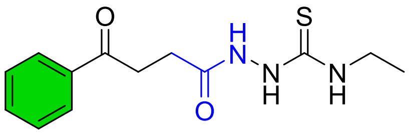	Mild Steel	0.5 mM	96	**4**
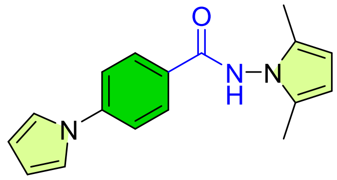	Mild Steel	0.5 mM	95	**5**
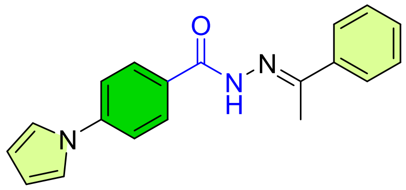	Mild Steel	0.5 mM	94	**6**

6. References.

1 1- Li, W.-h., He, Q., Zhang, S.-t., Pei, C.-l. & Hou, B.-r. Some new triazole derivatives as inhibitors for mild steel corrosion in acidic medium. *J. Appl. Electrochem.* **38**, 289-295 (2008). https://doi.org:10.1007/s10800-007-9437-7

2 Al-Edan, A. K. *et al.* Palmitic acid-based amide as a corrosion inhibitor for mild steel in 1M HCl. *Heliyon* **9**, e14657 (2023). https://doi.org:https://doi.org/10.1016/j.heliyon.2023.e14657

3 Alamiery, A. A. Investigations on corrosion inhibitory effect of newly quinoline derivative on mild steel in hcl solution complemented with antibacterial studies. *Biointerface Research in Applied Chemistry* **12**, 1561-1568 (2022). https://doi.org:10.33263/BRIAC122.15611568

4 Alamiery, A. Short report of mild steel corrosion in 0.5 m H2SO4 by 4-ethyl-1-(4-oxo-4-phenylbutanoyl)thiosemicarbazide. *Jurnal Tribologi* **30**, 90-99 (2021).

5 Alamiery, A. A. Anticorrosion effect of thiosemicarbazide derivative on mild steel in 1 M hydrochloric acid and 0.5 M sulfuric Acid: Gravimetrical and theoretical studies. *Materials Science for Energy Technologies* **4**, 263-273 (2021). https://doi.org:https://doi.org/10.1016/j.mset.2021.07.004

6 A.A. Alamiery, W.N.R.W. Isahak, H.S.S. Aljibori, H.A. Al-Asadi, A.A.H. Kadhum, Effect of the structure, immersion time and temperature on the corrosion inhibition of 4-pyrrol-1-yl-n-(2,5-dimethyl-pyrrol-1-yl)benzoylamine in 1.0 m HCl solution, Int. J. Corros. Scale Inhib. 10 (2) (2021) 700–713, https://doi.org/ 10.17675/2305-6894-2021-10-2-14.
